# Effects of acute and chronic gamma irradiations on regeneration of tissue types from selected Ugandan cassava genotypes

**DOI:** 10.3389/fpls.2025.1726057

**Published:** 2026-02-26

**Authors:** Hellen B. Apio, Emmanuel Ogwok, Norazlina Noordin, Peter Wasswa, Isaac Kofi Bimpong, Titus Alicai, Settumba B. Mukasa, John Odipio

**Affiliations:** 1 College of Agricultural and Environmental Sciences, Makerere University, Kampala, Uganda; 2 National Agricultural Research Organisation (NARO), National Crops Resources Research Institute (NaCRRI), Kampala, Uganda; 3 Department of Science and Vocational Education, Faculty of Education, Lira University, Lira, Uganda; 4 Agrotechnology and Biosciences Division, Malaysian Nuclear Agency, Bangi, Kajang Selangor, Malaysia; 5 Joint Plant Breeding and Genetics Section Joint Food and Agricultural Organisation (FAO)/ International Atomic Energy Agency (IAEA) Centre of Nuclear Techniques in Food and Agriculture, Department of Nuclear Sciences and Applications International Atomic Energy Agency, Vienna International Centre, Vienna, Austria

**Keywords:** cassava genotypes, tissue types, acute and chronic gamma irradiations, Growth reduction lethal dose (GR50), regeneration

## Abstract

Acute and chronic gamma irradiations (ACGIs) has facilitated generation of new varieties that can withstand abiotic and biotic stresses. This study examined the impact of ACGI doses on the regenerative ability of in vitro embryogenic tissues into complete plants. Tissues from nine genotypes (Alado, NASE 13, NASE 19, NAROCASS 1, CV_193, CV_98/0505, CV_60444, BAO, and reference Ubi Putih) were subjected to acute gamma irradiation (AGI) across six dose levels (0, 5, 10, 15, 20, 25, 35 Gy). Five genotypes (Ubi Putih, CV_60444, Alado, NASE 13, NAROCASS 1) were exposed to chronic gamma irradiation (CGI) for one to four weeks. Ten explants per genotype per dose were evaluated. Mean shoot length was used to calculate the growth reduction dose (GR50), and cell structure changes were analyzed using hematoxylin and eosin staining. Exposure of tissues to AGI and CGI enhanced shoots, roots, leaf regeneration as well as shoot length at dose rates of 5-20 Gy (AGI) and 46 Gy (CGI) at twoweeks. The calculated GR50 varied across genotypes and irradiation types. Histological analysis confirmed that distinct structural changes in cell and tissue morphology were linked to irradiation exposure. These results demonstrate that ACGIs can induce accelerated regeneration and structural variation in embryogenic cassava tissues, revealing the potential to develop novel varieties with improved stress resilience. The study underscores ACGIs as a viable biotechnological tool in mutation breeding and genetic improvement of root crops.

## Introduction

Cassava (*Manihot esculenta* Crantz) is a vital diploid crop (2n = 36) (Ceballos et al., 2015). Renowned for its resilience, cassava thrives in harsh climatic conditions and low rainfall regions, making it a reliable food security crop. Cassava stakes are utilized for animal feed, its flour serves as a supplement to wheat flour in the confectionery industry, and its starch plays a critical role in pharmaceutical production, therefore reinforcing its economic importance (Fu, 2021). Despite its significance, cassava faces serious threats from viral diseases, particularly cassava mosaic disease (CMD) and cassava brown streak disease (CBSD), with CBSD capable of causing total yield loss. Several cassava varieties bred for CMD resistance have succumbed to CBSD, however NAROCASS 1 remains the only exception, highlighting the narrow genetic pool available for future breeding programs (Kawuki et al., 2016; Nakabonge et al., 2018). To complement traditional breeding efforts and expand the genetic diversity of cassava with resistance to CBSD, researchers have proposed the integration of plant cell, tissue, and organ culture (PCTOC) techniques alongside induced mutagenesis using acute and chronic gamma irradiations (ACGIs), in an effort to develop varieties that benefit farmers, researchers and the public.

Plant cell, tissue, and organ culture (PCTOC) has been extensively utilized in fundamental research to investigate micropropagation, somatic embryogenesis, organogenesis, gene function, genetic transformation, and the development of homozygous pure lines (Loyola-Vargas and Ochoa-Alejo, 2024). Successful PCTOC applications rely on a robust plant regeneration system, which is governed by the cell’s totipotency, the ability to regenerate into a complete plant, and the tissue’s pluripotency, its capacity to differentiate into various cell types (Che et al., 2022).

Artificially induced mutations using physical mutagenic agents, particularly gamma rays, have been instrumental in generating genetic variability and developing improved crop varieties (Chaudhary et al., 2019). Gamma rays, categorized as low-linear energy transfer (LET) radiations, are widely used in mutation breeding. LET refers to the energy a particle or photon deposits per unit length as it traverses a material, and its value varies with the energy of the charged particle and the target species (Belfield et al., 2012; Russ et al., 2022). Gamma irradiation can be classified into acute exposure (high doses, >0.1Gy/min) or chronic exposure (low doses, <1Gy/hr over an extended period) (Hase et al., 2020).

Gamma radiation triggers genetic changes by causing primarily single-base substitutions (Shirasawa et al., 2016) and small insertions or deletions (InDels), resulting in genomic alterations within target tissues via the ionization of water molecules (Spencer-Lopes et al., 2018). The extent and nature of plant tissue responses to irradiation are influenced by multiple factors, including species, genotype, developmental stage, physiological and morphological traits, genome size, and inherent radiosensitivity (Lagoda, 2012; Datta, 2023). For instance, exposing cassava stakes to gamma irradiation doses of 25–30 Gy led to the development of the ‘Tebankye’ variety in Ghana, characterized by larger starch granules, improved cooking qualities, and resistance to African cassava mosaic disease (Yan et al., 2013). In Uganda, gamma irradiation of cassava genotypes, using both stakes and seeds, produced notable phenotypic changes, such as increased epicotyl length at higher radiation doses and reduced severity of CMD at 20 Gy and 15 Gy treatments (Baguma et al., 2021), additional the studies revealed that *in vitro* nodal cuttings (ivNCs) retained regenerative potential at doses ranging from 5–15 Gy, whereas friable embryogenic callus (FEC) experienced complete tissue death within 28 days post-irradiation (Apio et al., 2024).

Gamma radiation sources commonly used in mutation breeding include Cobalt-60 (Co-60) and Cesium-137 (Cs-137). Co-60 emits two gamma quanta (1.1715 MeV and 1.3316 MeV) and requires replacement every 15 years, making it costly. In contrast, Cs-137 continuously emits radiation with a single gamma ray (0.6614 MeV) and has a long half-life (30.17 years), reducing replacement costs (Grove, 1952). Exposure to ionizing radiation may result in either plant death or survival, depending largely on radiosensitivity, the capacity of cells, tissues, or whole organisms to withstand radiation. This trait significantly influences the survival and regeneration rates of mutant plants. In mutation breeding research, a critical metric is the lethal dose (LD50), defined as the radiation level that causes 50% mortality within a plant population (Fujii and MATSUMURA, 1958; Datta, 2023).

The explants examined included friable embryogenic callus (FEC), shoot apical meristems (SAMs), and *in vitro* nodal cuttings (ivNCs). SAMs, regions of active cell division and key sites for virus-free plant propagation, were used to assess irradiation effects on meristematic tissues. ivNCs, consisting of internodes with axillary buds capable of regenerating into whole plants, were evaluated for their regenerative capacity. FEC, a highly proliferative embryogenic system composed of totipotent single cells, served as a model for studying mutagenic responses under optimized *in vitro* conditions (Taylor et al., 1996; Apio et al., 2015). Tissue growth and development of the FEC, ivNCs and SAMs was modulated by plant growth regulators (PGRs), specifically benzylaminopurine (BAP) and naphthalene acetic acid (NAA), applied in controlled concentrations (Apio et al., 2015, Apio et al., 2021). This study investigated the effects of ACGI on the regenerative ability of the tissue types of different cassava genotypes once exposed to the different radiation doses.

## Materials and methods

### Experimental site and design

All experiments were conducted at the Malaysian Nuclear Agency, utilizing both the tissue culture laboratory and specialized irradiation facilities: the Acute Biobeam Gamma Cell Facility (ABGCF) and the Chronic Gamma Greenhouse (CGGH). A Completely Randomized Design (CRD) was employed to ensure unbiased treatment allocation and statistical robustness.

### Acute gamma irradiation (AGI) of ivNCs

Ten nodal cuttings (1.0-1.5 cm) were prepared for nine cassava genotypes (Ubi Putih, NASE 13, BAO, NASE 19, Alado, CV-60444, CV-193, CV-98/0505, NAROCASS 1) one day before irradiation and cultured in jars with containing Murashige and Skoog (MS) basal medium Murashige and Skoog (1962) supplemented with 30 g sucrose, 0.5 mL BAP, 0.1 mL NAA and 2.5 g gelrite. Jars were sealed with cling film to maintain sterility.

Gamma irradiation was performed using a Cesium-137 (Cs-137 chloride) pencil, using a Biobeam Gamma Cell Irradiator (BGCI) (Germany Service Medical GmbH, Leipzig). A Fricke dosimeter was placed at the center of the canister to verify accurate dose delivery. For each genotype and dose, two replicates of five explants were prepared totaling 10 explants per genotype per dose.

**Figure 1:**
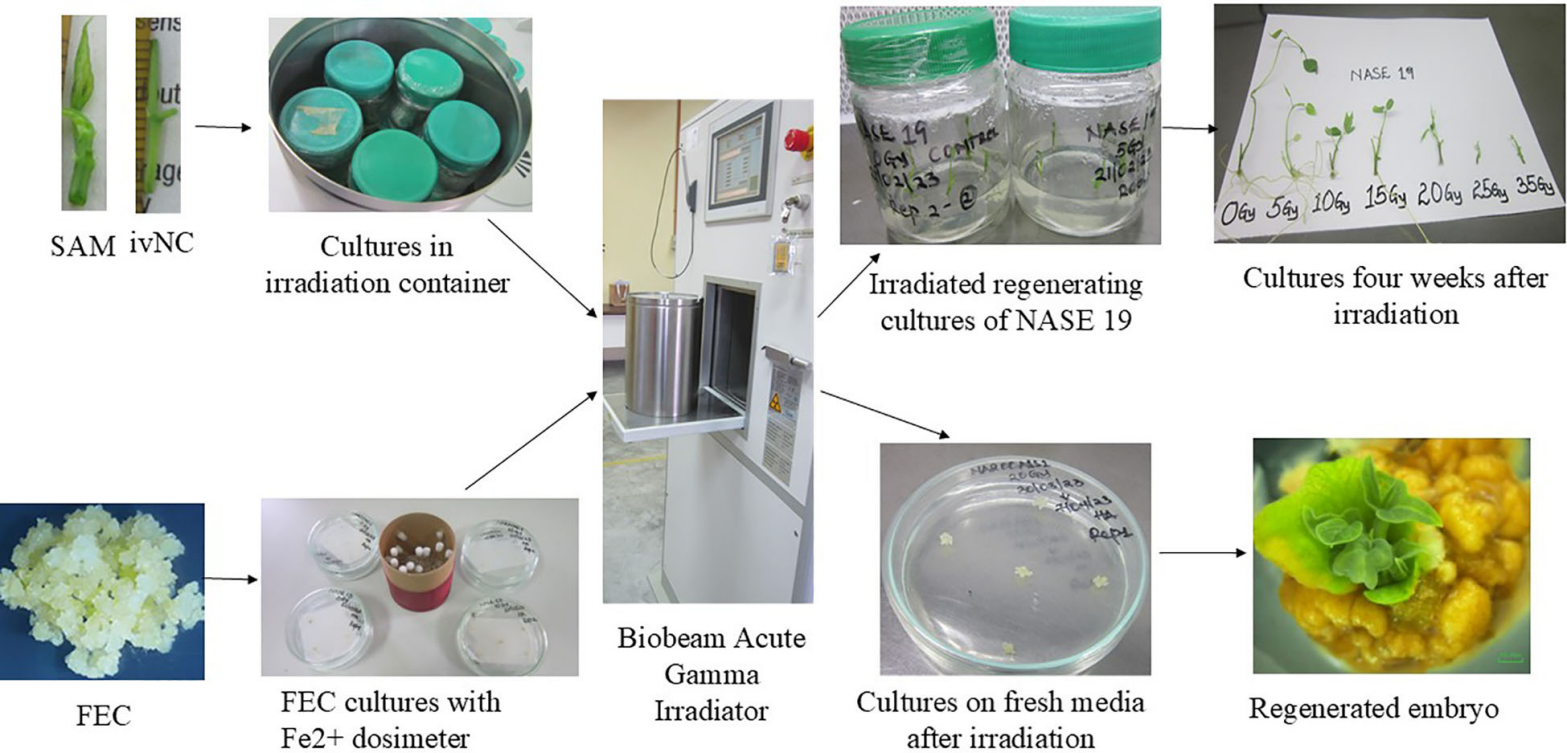
Schematic workflow illustrating acute gamma irradiation protocols for FEC, SAMs, and ivNCs under controlled in vitro conditions.

Explants were exposed to six irradiation doses (5, 10, 15, 20, 25, and 35 Gy) in the day, plus control (0 Gy) totaling 70 explants per genotype across treatments. Dose durations were calibrated as follows: 5 Gy: 1 min 32 sec; 10 Gy: 3 min 03 sec; 15 Gy: 4 min 35 sec; 20 Gy: 5 min 26 sec; 25 Gy: 6 min 58 sec; 35 Gy: 9 min 21 sec (**Figure 1**). Post-irradiation explants within the day were sterilized with 70% ethanol and transferred to a growth chamber maintained at 24 ± 2 °C under a 16-hour light/8-hour dark photoperiod. Weekly observations were recorded for four weeks, focusing on number of shoots, leaf count, root formation, shoot length. Data were analyzed using R version 4.4.2 and curve fit software to ascertain the growth lethal dose (GR50).

### AGI of SAMs

Ten cuttings of SAMs (1.0 cm) were prepared one day before irradiation and cultured in MS medium with the same supplements and sterilization protocol as above. This experiment was conducted on two genotypes: NASE 13 and Alado. For each genotype and dose, two replicates of five explants were prepared, totaling 10 explants per genotype per dose and 70 explants per genotype across all treatments (**Figure 1**).

Irradiation was performed using the same Cs-137 source and BGCI system, with dose durations identical to those used for nodal cuttings. After irradiation, explants were sterilized and placed in the growth room under the same environmental conditions. Due to limited explant availability, not all genotypes were included. Weekly data collection over four weeks focused on shoot number, leaf count, root formation, and shoot length. Analysis was conducted using R version 4.4.2 and curve fit software to ascertain the growth lethal dose (GR50).

### AGI of FEC

Ten clusters of FEC (approx. 0.4 × 0.4 mm²) were blotted on sterile Whatman paper to remove excess moisture and air-dried for 5 minutes in a laminar flow hood. Clusters were transferred to 90 × 15 mm sterile petri dishes containing a mesh overlay on MS medium supplemented with 30 g sucrose, 0.6 mL BAP, 3 mL NAA, and 3 g gelrite. Petri dishes were sealed with cling film to maintain sterility. This procedure was applied to two genotypes, NASE 13 and NAROCASS 1. For each genotype and dose, two replicates of five clusters were prepared, totaling 10 clusters per genotype per dose and 30 clusters per genotype across treatments. Gamma irradiation was performed using the same Cs-137 source and BGCI system, with doses of 0, 10, and 20 Gy. Exposure times were 10 Gy: 3 min 03 sec and 20 Gy: 5 min 26 sec (**Figure 1**). After irradiation, explants were sterilized and transferred to the growth room under the same conditions. Only genotypes capable of producing FEC were included. Cultures were monitored for survival, embryo formation, and plant regeneration. Data were collected at four weeks post-irradiation, measuring root number, leaf count, shoot formation, and shoot length. Statistical analysis was performed using R version 4.4.2 and curve fit software to ascertain the growth lethal dose (GR50).

### Chronic gamma irradiation (CGI) of cassava explant under controlled conditions

**Figure 2:**
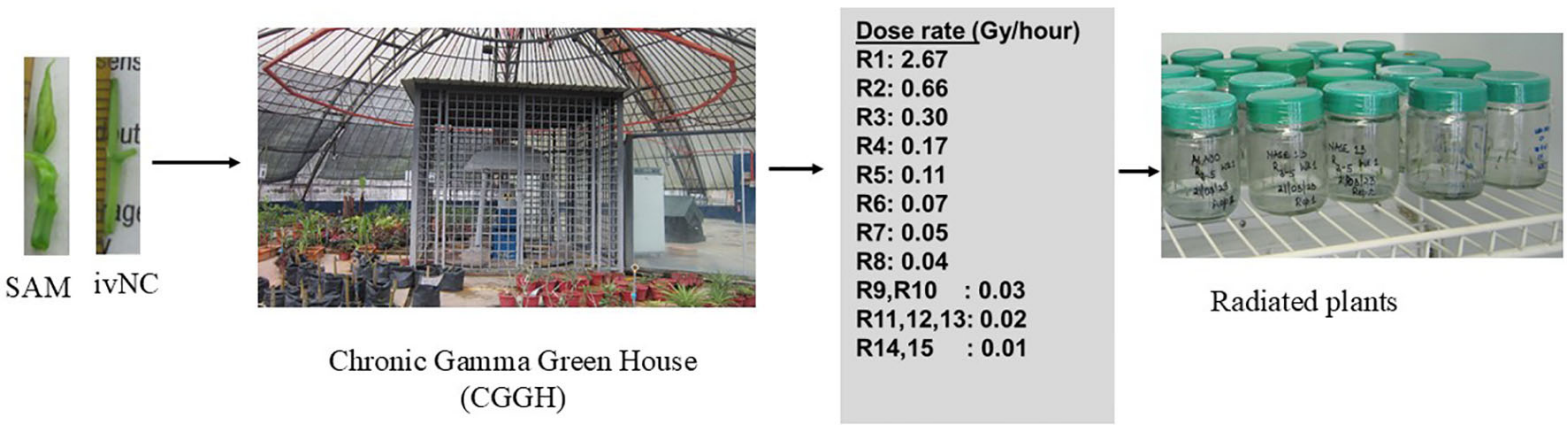
Workflow outlining chronic irradiation methodology applied to SAMs and ivNCs under controlled *in vitro* conditions.

The Chronic Gamma Greenhouse (CGGH) was specifically designed to facilitate long-term gamma irradiation experiments. It is shielded by a 300-meter natural barrier of trees and enclosed by a protective wall measuring 2 meters in height and 1 meter in width. This setup ensures environmental containment and safety during irradiation procedures (**Figure 2**).

For the study of radiosensitivity of ivNCs, ten nodal explants, each 1.0 cm in length, were prepared one day prior to irradiation. These were cultured in sterile jars containing MS basal medium, supplemented with 30 g sucrose, 0.5 mL BAP, 0.1 mL NAA, and 2.5 g gelrite. The jars were sealed with cling film to maintain sterility. This procedure was applied to four cassava genotypes: Ubi Putih, NASE 13, Alado, and CV-60444. For each genotype and dose per week, two replicates of five explants were prepared, resulting in 10 explants per genotype per week. Over the four-week CGI period, this totaled 40 explants per genotype and 160 explants across all genotypes. Explants were placed at ring position three within the CGGH, which emits gamma radiation at a rate of 0.3 Gy per hour, a setting optimized for cassava growth.

The irradiation source was a Cesium-137 (Cs-137 chloride) pencil, shielded by lead. Radiation doses were calculated based on exposure duration: Week 1 involved 3.79 days of exposure (27.34 Gy), Week 2 had 6.44 days (46.37 Gy), Week 3 lasted 9.5 days (68.65 Gy), and Week 4 extended to 11 days (79 Gy). After each weekly exposure, explants were transferred to the tissue culture laboratory, surface-sterilized with 70% ethanol to eliminate residual contamination, and moved to a growth room maintained at 24 ± 2 °C for 16-hour light and 8-hour dark photoperiod. Data collection focused on survival rate, number of shoots, number of leaves, number of roots, and shoot length. These parameters were recorded weekly for four weeks post-irradiation to monitor growth progression. The collected data were analyzed using R version 4.4.2.

In a parallel experiment, radiosensitivity of SAMs was assessed using a similar protocol. Ten SAM explants, each 1.0 cm in length, were prepared and cultured in MS medium with the same supplements and sterilization procedures. This experiment was conducted for two cassava genotypes: NASE 13 and Alado. Two replicates of five explants were prepared per genotype per dose per week, resulting in 10 explants per genotype per week. Over the three-week CGI period, this totaled 30 explants per genotype and 60 explants overall.

Explants were irradiated at ring position three in the CGGH, using the same Cs-137 pencil source. Radiation doses were calculated as follows: Week 1 involved 2.81 days of exposure (21.23 Gy), Week 2 had 3.38 days (27.33 Gy), and Week 3 lasted 4.41 days (31.79 Gy). After each exposure period, explants were sterilized with 70% ethanol and transferred to the growth room under identical environmental conditions. Observations for SAM explants were recorded weekly for three weeks post-irradiation, focusing on survival rate, shoot number, leaf number, root number, and shoot length. Data were analyzed using R version 4.4.2 to evaluate the effects of chronic gamma irradiation on meristematic tissue development.

### Calculation of growth reduction lethal dose (GR_50_)

Data on key growth parameters including number of leaves, roots, shoots produced, and shoot length were documented weekly for four consecutive weeks following irradiation. Mean shoot length was selected as the primary parameter based on the *in vitro* response of different cultures, serving as the basis for calculating the 50% (GR_50_) growth reduction lethal doses. Under the laminar flow hood, the shoot length of each plant was measured weekly using a meter ruler to assess growth progression. Data for each genotype across different irradiation doses were systematically recorded in Microsoft Excel 2010. Mean values corresponding to the applied doses were calculated to establish the relationship between the independent variable (dose) and the dependent variable (shoot length), aiding in the determination of the best-fit polynomial regression model. The GR_50_ values were derived using the linear regression equation:

Y=mX+bWhere:

- Y represents the dependent variable (shoot length).
- X denotes the independent variable (dose)
- m is the estimated slope and
- b is the estimated intercept

This analysis was conducted separately for the different tissue types: ivNCs, SAMs and FEC and across both acute and chronic radiation treatments.

### Histological observations of cassava mutant lines

Cassava mutant lines regenerated from FEC, SAMs, and ivNCs, subjected to varying doses of gamma irradiation along with non-irradiated controls, were collected for histological analysis. Samples were fixed in formalin-acetic acid-alcohol (FAA) solution for 12 hours at room temperature (25 °C) using an automatic tissue processor (LEICA TP 1020, Germany). Following fixation, the samples were embedded in paraffin wax using a moulding unit (MEDAX GmbH & CO., Germany). Thin sections of 15 µm thickness were cut using a manual rotary microtome (LEICA RM 2235, Germany) and mounted on standard glass slides. The slides were dried overnight at 63 °C in an Esco Isotherm oven. Once dried, the sections were deparaffinized in xylene for 20 minutes, then rehydrated sequentially in 100% ethanol (1–2 minutes) followed by 95% ethanol (1–2 minutes). The rehydrated sections were rinsed thoroughly in tap water and distilled water before staining.

Staining was performed using hematoxylin (LOBA CHEMIE PVT LTD) for 3–5 minutes, followed by differentiation using hydrochloric acid in 70% ethanol (two quick dips). The slides were then rinsed in running tap water for 15 minutes and counterstained with eosin for up to 4 minutes. Post-staining, the slides were dehydrated and differentiated by dipping six times in 95% ethanol, followed by six dips in 100% ethanol. The sections were then cleared twice in xylene and mounted using 100% ethanol as the mounting medium for 3 minutes, following the histopathology protocol of the Veterinary Medicine Laboratory, Makerere University. Finally, the prepared slides were examined under a transmission electron microscope (Nikon CSI, Japan) at CDL Laboratories, Veterinary Medicine, Makerere University, and representative images were documented for analysis.

## Results

### Effects of AGI doses on the regeneration of ivNCs of different cassava genotypes

#### Effects of genotype on growth parameters

The response of ivNCs from nine cassava genotypes to AGI was assessed based on morphological growth indicators, including shoot length, number of shoots, leaves, and roots. Performance varied among genotypes, with optimal growth observed at radiation doses of 5–15 Gy, while a decline in growth parameters occurred at higher doses (20–35Gy) after the first regeneration stage (FRS).

**Figure 3:**
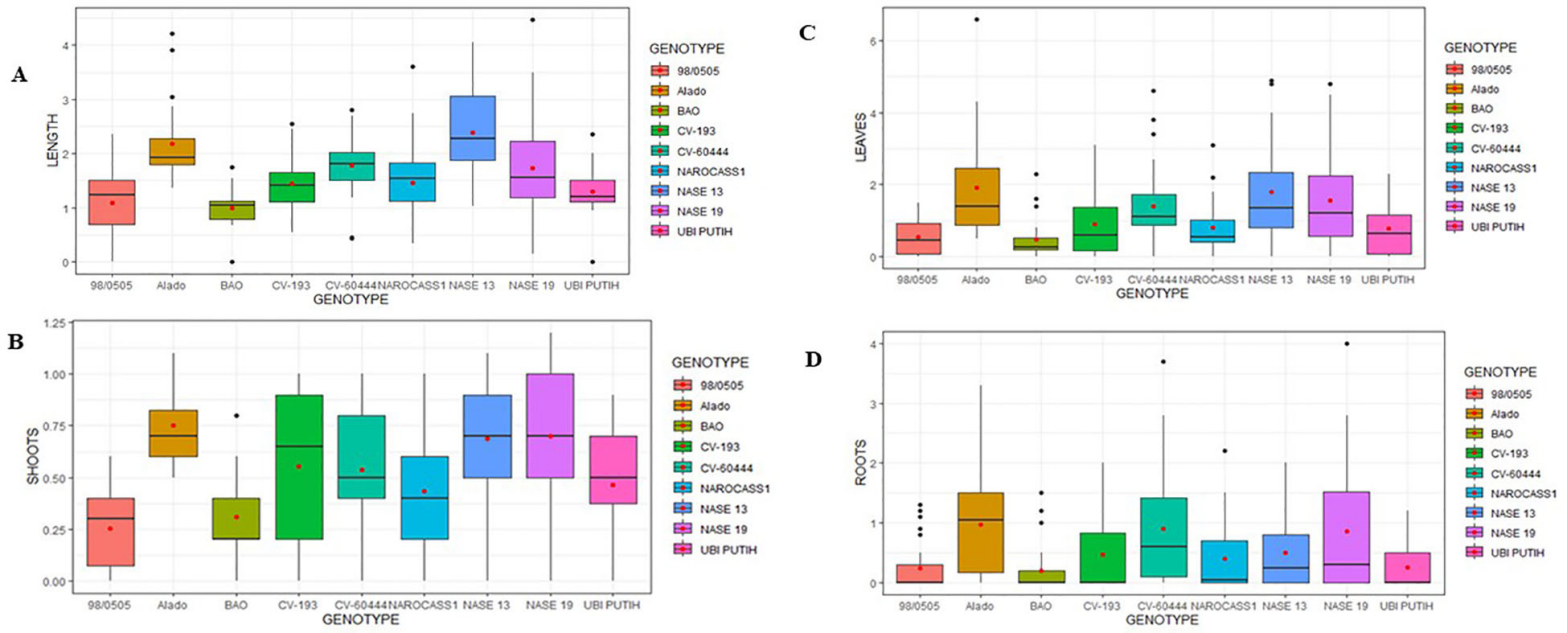
Mean values of key morphological parameters measured in ivNCs across genotypes over a four-week period following gamma irradiation. **(A)** Average shoot length per genotype. **(B)** Average number of shoots per genotype. **(C)** Average number of leaves per genotype. **(D)** Average number of roots per genotype.

*Shoot Length*: The highest mean shoot length after FRS, compared to the reference cultivar (Ubi Putih, 1.33 ± 0.21cm), was recorded in NASE 13 (2.38 ± 0.32cm), followed by Alado (2.18 ± 0.27cm), and the lowest in BAO (1.13 ± 0.24cm) (**Figure 3A**). Genotypic differences were statistically significant (P = 2.2 × 10^-16^) at 99.99% confidence interval.

*Number of Shoots*: The number of shoots varied across genotypes, with Alado (0.75 ± 0.14) producing the highest count, followed by NASE 19 (0.70 ± 0.20) and NASE 13 (0.69 ± 0.18). The lowest was observed in 98/0505 (0.26 ± 0.15) (**Figure 3B**). Genotypic differences were significant (P = 2.2 × 10^-16^) at 99.99% confidence interval.

*Leaf Production*: Alado exhibited the highest leaf production (1.91 ± 0.39), followed by NASE 13 (1.81 ± 0.38) and NASE 19 (1.5 ± 0.36), while BAO had the lowest (0.68 ± 0.31) (**Figure 3C**). Significant genotype differences were observed (P< 2 × 10^-16^) at 99.99% confidence interval.

Root formation varied significantly across genotypes. Alado exhibited the highest mean root count (0.97 ± 0.33), markedly outperforming the reference cultivar Ubi Putih (0.31 ± 0.21). NASE 19 (0.86 ± 0.36) and CV-60444 (0.84 ± 0.35) followed closely, while BAO recorded the lowest mean count (0.39 ± 0.28) (**Figure 3D**). Genotypic differences were statistically significant (P = 2.2 × 10^-16^) at a 99.99% confidence interval.

#### Effects of AGI doses on growth parameters

**Figure 4:**
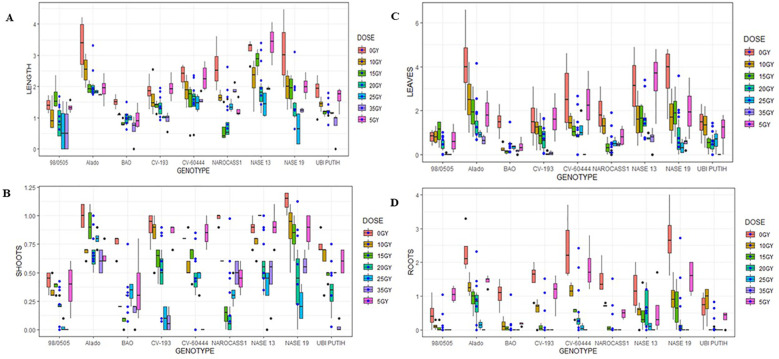
Mean values of morphological parameters measured in ivNCs across nine cassava genotypes following interaction with six gamma irradiation dose rates over a four-week period. **(A)** Average shoot length per genotype. **(B)** Average number of shoots per genotype. **(C)** Average number of leaves per genotype. **(D)** Average number of roots per genotype.

*In vitro* nodal cuttings (ivNCs) were exposed to six gamma radiation doses (5, 10, 15, 20, 25, and 35 Gy), revealing distinct genotype-specific responses. In comparison to the control 0Gy for each genotype, Alado performed best at 10Gy, NASE 13 at 5Gy, NASE 19 at 10Gy, NAROCASS 1 at 10Gy, BAO at 10 Gy, CV-193 at 5Gy, CV-60444 at 5 Gy and the control genotype Ubi Putih at 5Gy (**Figure 4**) for parameters measured.

*Shoot length*: The highest shoot length was recorded in NASE 13 at 5 Gy (3.4 ± 0.29 cm), followed by Alado at 10 Gy (2.52 ± 0.25 cm) and NASE 13 again at 15 Gy (2.83 ± 0.18 cm). A progressive decline in shoot elongation was observed beyond 20 Gy. At 35 Gy, BAO and 98/0505 exhibited the lowest shoot lengths (0.63 ± 0.22 cm and 0.63 ± 0.38 cm, respectively) (**Figure 4A**). Genotype-by-dose interactions were statistically significant (P = 2.2 × 10^-16^).

*Number of Shoots*: Genotypic differences were evident, with the highest values recorded in NASE 19 at 0 Gy (1.13 ± 0.05) and NASE 13 at 15 Gy (1.0 ± 0.00). At 35 Gy, CV-60444 and 98/0505 failed to produce shoots (0.0 ± 0.00) (**Figure 4B**). Significant genotype-dose interactions were observed (P = 2.2 × 10^-16^).

*Leaf Production:* The highest leaf production was recorded in Alado at 0 Gy (4.15 ± 0.95) and NASE 13 at 5 Gy (3.40 ± 0.73). Alado produced relatively high numbers at 10Gy (2.55 ± 0.67) and 15Gy (1.95 ± 0.49), 20Gy in NASE 13 (1.43 ± 0.26), 25Gy in CV-60444 (1.23 ± 0.26) and 35Gy in NASE 13 (0.7 ± 0.25) (**Figure 4C**). Ubi Putih, CV-60444 and 98/0505did not produce leaves at 35Gy. Significant differences due to genotype-dose interactions were noted (P = 2.96 × 10^-8^) at 99 %CI.

*Root Formation*: NASE 19 exhibited the highest root production at 0 Gy (2.73 ± 0.49), followed by CV-60444 at 5Gy (1.85 ± 0.35), 10Gy in Alado (1.30 ± 0.15), 15Gy in Alado (0.90 ± 0.18), 20Gy in Alado (0.70 ± 0.25), 25Gy in Alado (0.15 ± 0.06). All genotypes did not produce roots at 35 Gy (**Figure 4D**). No genotypes produced roots at 35 Gy (**Figure 4D**). Significant genotype-dose interactions were observed (P = 2.2 × 10^-16^).

The study revealed that acute gamma irradiation significantly influenced the regeneration potential of cassava ivNCs. The genotypes Alado and NASE 19 performed relatively well in relation to the parameters measured while BAO and 98/0505 were low. Optimal regeneration occurred at 5–15 Gy, while growth declined at 20–35 Gy, with complete inhibition at the highest dose for certain genotypes.

### Effects of AGI on ivNCs of nine cassava genotypes with respect to time in weeks

**Figure 5:**
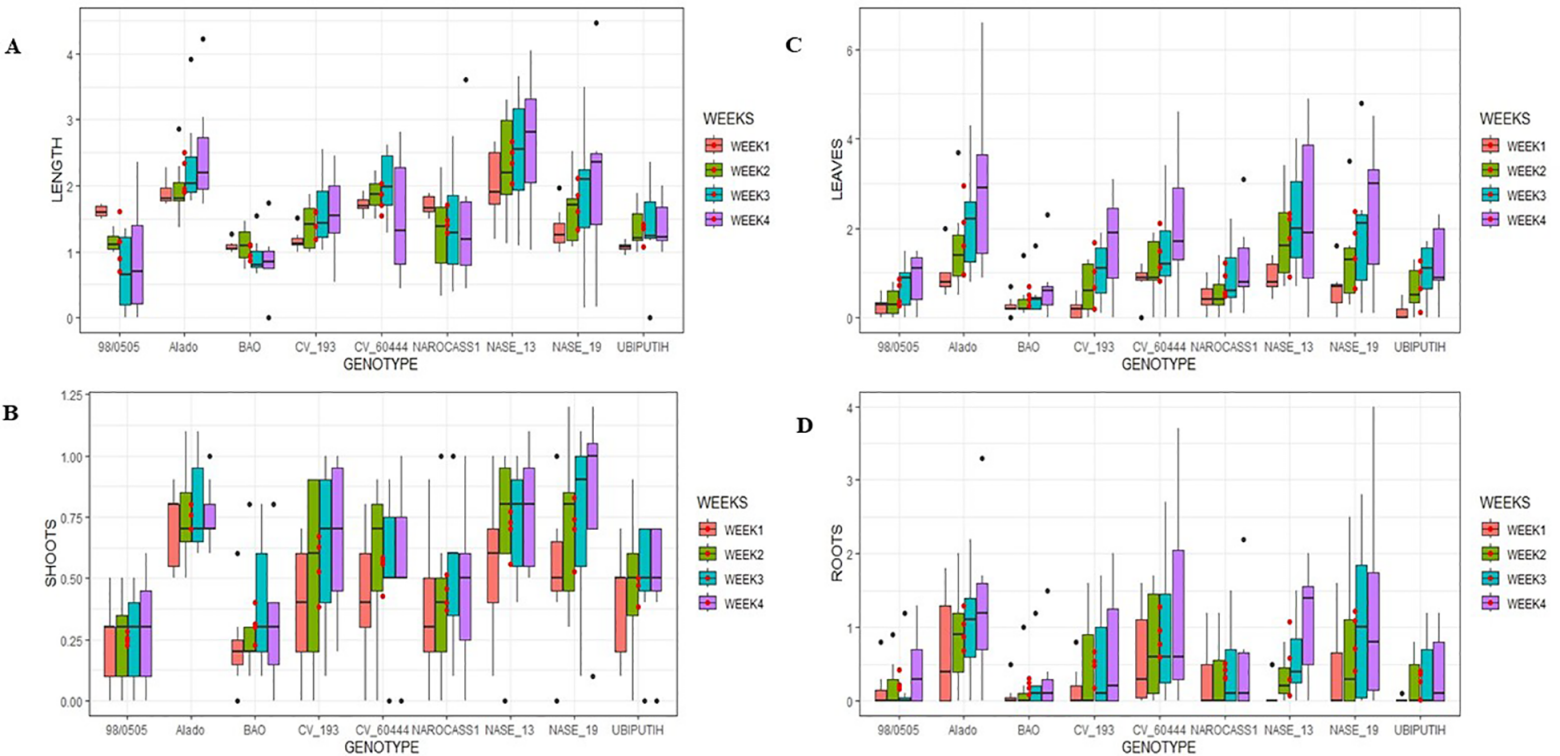
Mean values of morphological parameters measured in ivNCs across nine cassava genotypes over a four-week period, highlighting genotype-by-time interactions. **(A)** Average shoot length per genotype over time. **(B)** Average number of shoots per genotype over time. **(C)** Average number of leaves per genotype over time. **(D)** Average number of roots per genotype over time.

The ivNCs of nine cassava genotypes were subjected to six gamma irradiation doses (5, 10, 15, 20, 25 and 35 Gy), with data collected at weeks 1, 2, 3, and 4. The trends illustrated in **Figure 5** indicate that growth parameters including shoot length, number of shoots, number of leaves, and roots generally increased over time. However, in some genotypes, a decline in these parameters was observed by week 4.

*Shoot Length:* The mean shoot length of BAO, NAROCASS 1, and 98/0505 decreased progressively with time, whereas that of Alado, CV-193, CV-60444, NASE 13, and NASE 19 exhibited an increase in shoot length across the four-week period. While the effect of time alone did not yield significant differences (P = 0.266, 5% CI), the interaction between genotype and time was statistically significant (P = 0.006536, 99.9% CI). By the fourth week, NASE 13 recorded the highest mean shoot length, followed by NASE 19, while 98/0505 had the lowest (**Figure 5A**).

*Number of Shoots:* The mean number of shoots produced varied among genotypes. In 98/0505 and Ubi Putih, shoot production remained unchanged over time. In contrast, BAO, CV-193, CV-60444, NASE 13, and NASE 19 exhibited a gradual increase in shoot production throughout the four weeks. NASE 19 produced the highest number of shoots, followed by NASE 13, CV-193, and BAO, which had the lowest. The effect of time alone was statistically significant (P = 3.956 × 10^-5^, 99.999% CI); however, genotype-time interaction showed no significant differences (P = 0.9965, 5% CI) (**Figure 5B**).

*Number of Leaves:* Leaf production varied across genotypes. A gradual increase in the average number of leaves was observed for Alado, 98/0505, CV-193, BAO, CV-60444, NAROCASS 1, and NASE 19 over the four-week period. However, NASE 13 and Ubi Putih initially showed an increase before declining in week 4. At week 4, NASE 19 exhibited the highest leaf production, followed by Alado and CV-193, while BAO recorded the lowest. The effect of time alone was highly significant (P< 2 × 10^-16^, 99.999% CI), though genotype-time interaction was not statistically significant (P = 0.4904, 5% CI) (**Figure 5C**).

*Number of Roots:* Root production varied among genotypes. A gradual increase in the average number of roots was observed for Alado, 98/0505, CV-193, BAO, CV-60444, NAROCASS 1, and Ubi Putih throughout the four weeks. NASE 13 exhibited a progressive increase initially, followed by exponential growth between weeks 3 and 4. Conversely, NASE 19 showed a steady increase in root production before declining at week 4. By week 4, NASE 13 recorded the highest root production, followed by Alado and NASE 19, with BAO producing the lowest. The effect of time alone was statistically significant (P = 2.402 × 10^-10^, 99.999% CI), while genotype-time interaction showed no significant differences (P = 0.8367, 5% CI) (**Figure 5D**).

The study demonstrated that gamma irradiation influenced cassava ivNC regeneration differently across genotypes. While growth parameters generally increased over time, some genotypes exhibited a decline by week 4. Genotype-time interaction had a statistically significant effect on shoot length but was not significant for shoot, leaf, or root production.

### Effects of AGI on SAMs regeneration in alado and NASE 13

The SAMs of two cassava genotypes (Alado and NASE 13) were subjected to gamma irradiation at six doses (5, 10, 15, 20, 25 and 35 Gy) alongside a control (0 Gy). Increasing radiation dose generally led to a decline in shoot length, number of shoots, roots, and leaves, though lower doses occasionally promoted growth before reductions occurred at higher doses.

**Figure 6:**
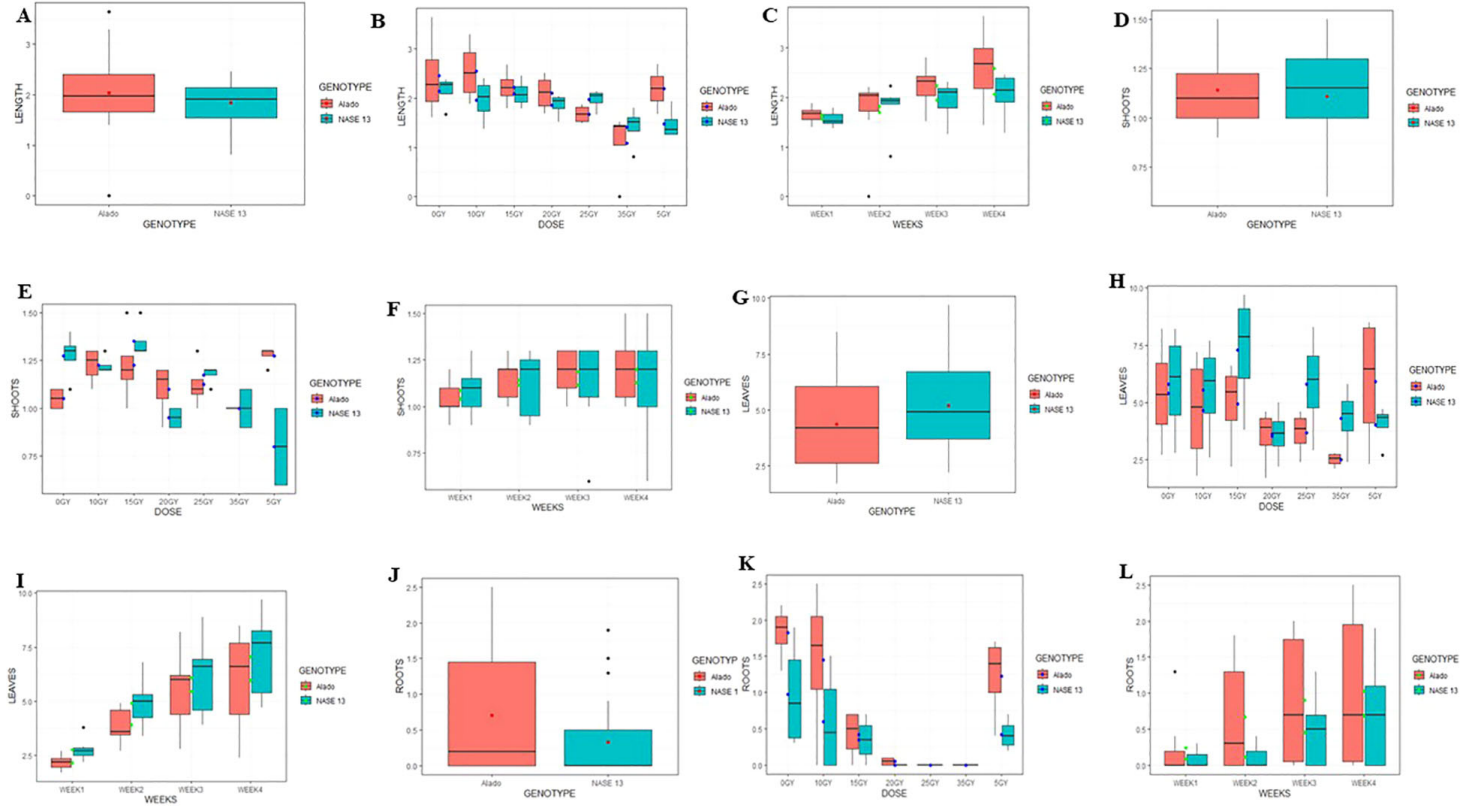
Mean values of morphogenic parameters in SAMs of cassava genotypes Alado and NASE 13 following exposure to six AGI. dose rates over a four-week culture period. **(A)** Average shoot length per genotype. **(B)** Shoot length variation due to genotype × dose rate interaction. **(C)** Shoot length variation due to genotype × time (weeks) interaction. **(D)** Average number of shoots per genotype. **(E)** Shoot count variation due to genotype × dose rate interaction. **(F)** Shoot count variation due to genotype × time (weeks) interaction. **(G)** Average number of leaves per genotype. **(H)** Leaf count variation due to genotype × dose rate interaction. **(I)** Leaf count variation due to genotype × time (weeks) interaction. **(J)** Average number of roots per genotype. **(K)** Root count variation due to genotype × dose rate interaction. **(L)** Root count variation due to genotype × time (weeks) interaction.

*Shoot Length:* Mean shoot length at the FRS varied between genotypes: Alado (1.97 ± 0.28cm) outperformed NASE 13 (1.78 ± 0.20cm), though both controls (Alado: 2.45 ± 0.44cm, NASE 13: 2.15 ± 0.16cm) exhibited better growth than irradiated plants. Alado consistently outperformed NASE 13, with significant genotype differences (P = 0.039, 95% CI) (**Figure 6A**). At 10 Gy, Alado showed increased shoot length (2.55 ± 0.31 cm) compared to its control (2.45 ± 0.44cm), while NASE 13 performed best at 15 Gy (2.05 ± 0.18cm). Significant differences were observed for shoot length across doses (P = 3.22 × 10^-6^, 99.999% CI) and genotype-dose interaction (P = 0.024, 95% CI) (**Figure 6B**). Genotype-week interaction showed progressive shoot length increase in both genotypes, with Alado maintaining superior growth at week four (P = 1.622 × 10^-5^, 99.999% CI). However, genotype-week interaction was not significant (P = 0.1328, 95% CI) (**Figure 6C**).

*Number of Shoots:* Mean shoot counts at the FRS exhibited minor variation between genotypes. Alado recorded 1.16 ± 0.13 shoots, while NASE 13 produced 1.09 ± 0.18 shoots, compared to their respective controls (NASE 13: 1.28 ± 0.06; Alado: 1.05 ± 0.03). These differences were not statistically significant (P = 0.2949; 95% CI) (**Figure 6D**). However, radiation dose exerted a pronounced effect: Alado responded positively at 5 Gy (1.28 ± 0.03), surpassing its control, whereas NASE 13 achieved its highest shoot count at 15 Gy (1.35 ± 0.05). Dose-dependent variation was statistically significant (P = 2.337 × 10^-5^; 99.999% CI), as was the genotype-by-dose interaction (P = 1.105 × 10^-5^; 99.999% CI) (**Figure 6E**). Temporal analysis revealed a progressive increase in shoot production up to week two, with no further gains observed in weeks three and four (P = 0.3790; 95% CI). Genotype-by-week interaction was also non-significant (P = 0.7590; 95% CI) (**Figure 6F**), suggesting a plateau in shoot induction kinetics beyond the early culture phase.

*Number of Leaves:* Mean leaf production varied between genotypes. Alado recorded 4.37 ± 0.39 leaves, while NASE 13 produced 5.05 ± 0.44 leaves compared to their controls (NASE 13: 5.80 ± 1.20, Alado: 5.40 ± 1.12). Significant genotypic differences were observed (P = 0.0011, 99.99% CI) (**Figure 6G**). At 5 Gy, Alado showed an increase (5.93 ± 1.48) compared to its control (5.40 ± 1.12), while NASE 13 performed best at 15 Gy (7.30 ± 1.32) compared to its control (5.80 ± 1.20). Significant differences were observed for dose-dependent effects (P = 8.201 × 10^-7^, 99.999% CI) and genotype-dose interaction (P = 0.0002518, 99.999% CI) (**Figure 6H**). Weekly progression showed a steady increase, with NASE 13 consistently outperforming Alado (P = 3.767 × 10^-11^, 99.999% CI). Genotype-week interaction was not significant (P = 0.9115, 95% CI) (**Figure 6I**).

*Number of Roots*: Mean root production varied between Alado (0.97 ± 0.39) and NASE 13 (0.34 ± 0.22), compared to their controls (NASE 13: 0.98 ± 0.38, Alado: 1.83 ± 0.19). Significant genotypic differences were observed (P = 0.001149, 99.99% CI) (**Figure 6J**). At 5 Gy, Alado exhibited increased root formation (3.00 ± 0.89), while NASE 13 showed better root development at 10 Gy (0.68 ± 0.37). Root formation was observed at 5–20 Gy, but beyond 20 Gy, neither genotype formed roots. Significant differences were observed for dose-dependent effects (P = 2.276 × 10^-10^, 99.999% CI) and genotype-dose interaction (P = 0.0227, 95% CI) (**Figure 6K**). Weekly analysis showed progressive root development up to week two, with no further increase afterward (P = 2.90 × 10^-4^, 99.999% CI). Genotype-week interaction was not significant (P = 0.6184, 95% CI) (**Figure 6L**).

Acute gamma irradiation significantly affected SAM regeneration in cassava genotypes Alado and NASE 13, with low-to-moderate doses (5–15Gy) enhancing growth parameters, while higher doses (20–35Gy) inhibited development. Weekly progression indicated gradual improvements in shoot length, shoots, leaves, and roots, though genotype-week interactions did not yield statistically significant effects.

### Effects of gamma irradiation on FEC regeneration in NAROCASS 1 and NASE 13

**Figure 7:**
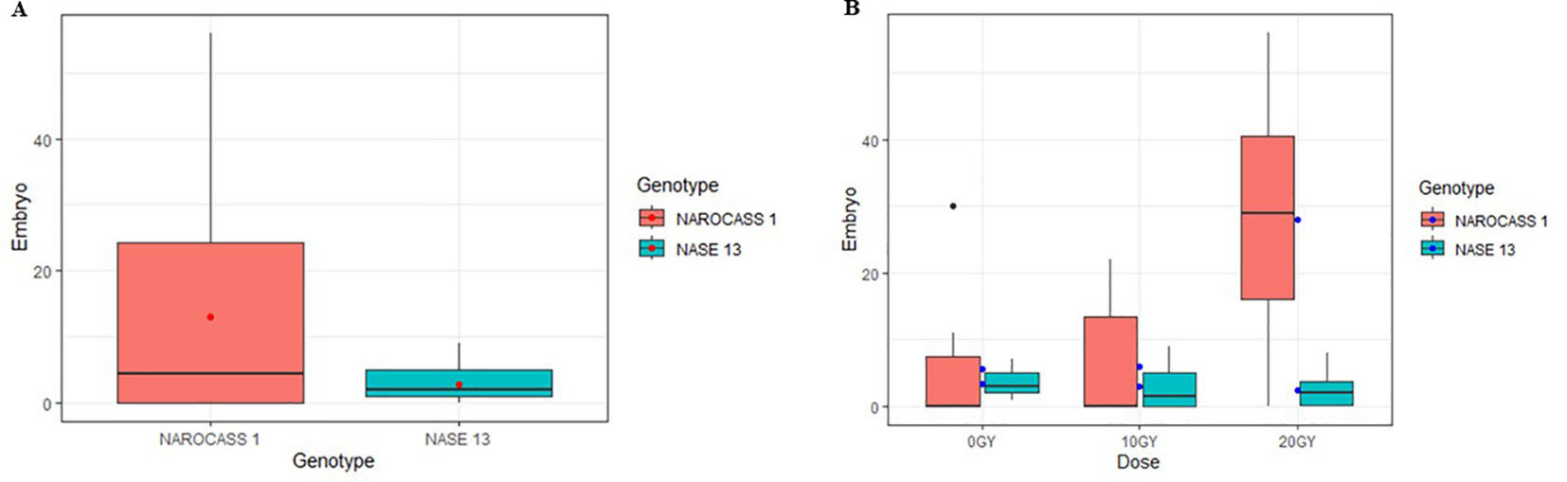
Embryogenic response of FEC from cassava genotypes Alado and NASE 13 following exposure to AGI. **(A)** Mean number of somatic embryos produced per genotype across all treatments. **(B)** Mean number of embryos generated from FEC after exposure to two radiation doses (10Gy and 20Gy).

Exposure of FEC from cassava genotypes NAROCASS 1 and NASE 13 to 10 Gy and 20 Gy of gamma irradiation, alongside a control (0 Gy), resulted in distinct genotype responses. NAROCASS 1 exhibited a higher number of regenerated embryos compared to NASE 13 (**Figure 7A**). The greatest embryo germination was observed at 20 Gy, surpassing that of 10 Gy in both genotypes (**Figure 7B**). For NAROCASS 1, the highest embryo count was recorded at 20 Gy (28.0 ± 5.78), followed by 10 Gy (5.90 ± 3.02) and the control 0 Gy (5.50 ± 3.02). Conversely, NASE 13 exhibited higher embryo germination at 0 Gy (3.40 ± 0.65) compared to 10 Gy (2.90 ± 1.06) and 20 Gy (2.40 ± 0.79). Statistical analysis showed no significant differences in embryo numbers across genotypes (P = 0.315) or radiation doses (P = 0.534) at 5% confidence interval.

**Figure 8:**
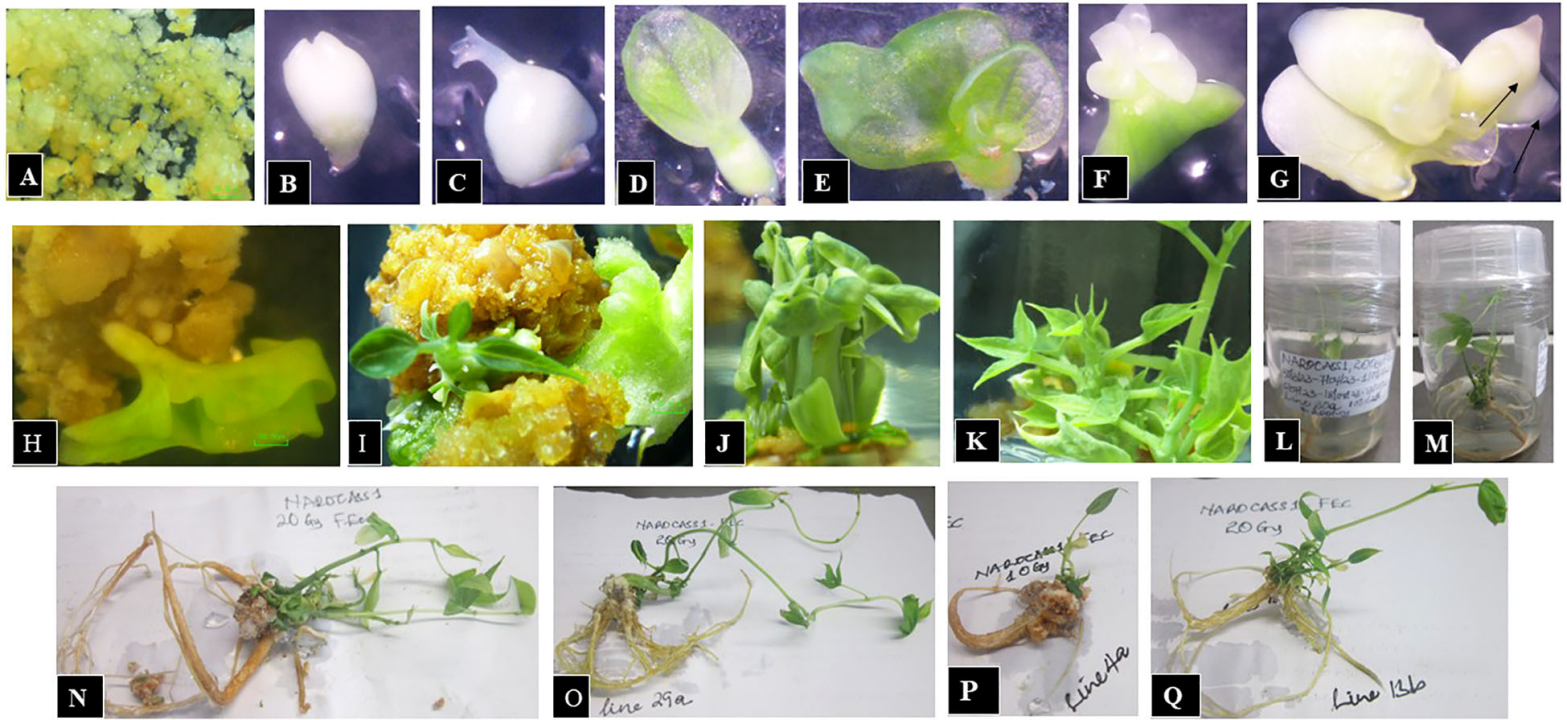
Sequential stages of somatic embryo development and regeneration from irradiated FEC of cassava genotype NAROCASS 1 following AGI. **(A)** Irradiated FEC. **(B)** Isolated dividing embryogenic cells exhibiting loss of pigmentation. **(C)** Discoloured embryo with emerging cotyledon. **(D)** Germinated embryo with one green cotyledon. **(E)** Germinated embryo with two green cotyledons. **(F)** Germinated embryo with one discoloured cotyledon and one green cotyledon. **(G)** Discoloured germinated embryo with two cotyledons and two distinct meristematic poles (indicated by arrows). **(H)** Fully germinated cotyledon-stage embryos post-irradiation. **(I)** Sprouting irradiated plantlet emerging from embryo with visible shoot and leaf structures. **(J, K)** Fully germinated mutant lines showing morphological variation attributable to irradiation. **(L, M)** Well-developed mutant cassava lines regenerated in vitro within culture jars. **(N-Q)** Fully regenerated mutant cassava lines exhibiting robust morphological systems following exposure to 10Gy and 20Gy.

To evaluate regeneration potential, the FEC of NAROCASS 1 and NASE 13 was cultured on media supplemented with NAA and BAP. The presence of these plant growth regulators facilitated asexual reproduction, exploiting mitotic division to generate genetically identical cells. This allowed embryonic development through key stages—torpedo, heart-shaped, cotyledonary, and ultimately mature plant formation (**Figures 8A-N**). While NAROCASS 1 successfully progressed through all developmental stages, NASE 13 failed to regenerate into whole plants, indicating a genotype-dependent response to irradiation and culture conditions.

### Dose-dependent morphogenic responses in regenerated cassava mutant lines

The vigor and developmental outcomes of regenerated mutant cassava lines varied significantly across radiation doses, as summarized in **Table 1**. Plants exposed to 20 Gy exhibited the greatest mean shoot length, indicating enhanced elongation potential at higher irradiation levels. Conversely, mutant lines treated with 10 Gy produced a higher number of shoots, suggesting that lower doses favor meristematic proliferation. Foliar development was more pronounced at 20 Gy, with a greater number of leaves observed compared to the 10 Gy treatment. These contrasting trends underscore a dose-dependent trade-off between shoot proliferation and leaf expansion, with lower doses promoting shoot initiation and higher doses enhancing vegetative growth.

**Table 1:** Morphogenic performance of mutant lines from irradiated FEC of NAROCASS 1.

Mutant line#	Dose	* Shoot length (cm)	*Numbers of Shoots	*Numbers of Leaves	*Numbers of Roots
1	10	2.20±0.31	04	08	1
2	20	2.05±0.55	02	11.5	0
3	20	2.60	01	10	0
4	20	1.80	01	10	3
5	20	1.84±0.87	05	4.5	0
6	20	2.60	01	10	2
7	20	2.00	01	13	0
8	20	1.80	01	06	0
9	20	2.00	01	06	2
10	20	1.60	01	05	0

* Mean Shoot length and number of shoots, leaves, roots from Regenerated Mutant lines of NAROCASS1 irradiated FEC.

Collectively, these findings highlight the importance of fine-tuning irradiation protocols to optimize morphogenic vigor and agronomic traits in cassava mutant lines. Strategic dose selection may enable targeted enhancement of specific developmental parameters, thereby improving the efficiency of mutation breeding and *in vitro* regeneration systems.

### Effects of CGI on ivNCs of four cassava genotypes

The *iv*NCs of four cassava genotypes (Alado, NASE 13, CV_60444, and Ubi Putih) were exposed to CGI at four doses (27, 46, 69 and 79 Gy) over four weeks. The cumulative effect of irradiation influenced shoot length, number of shoots, leaves, and roots differently across genotypes.

**Figure 9:**
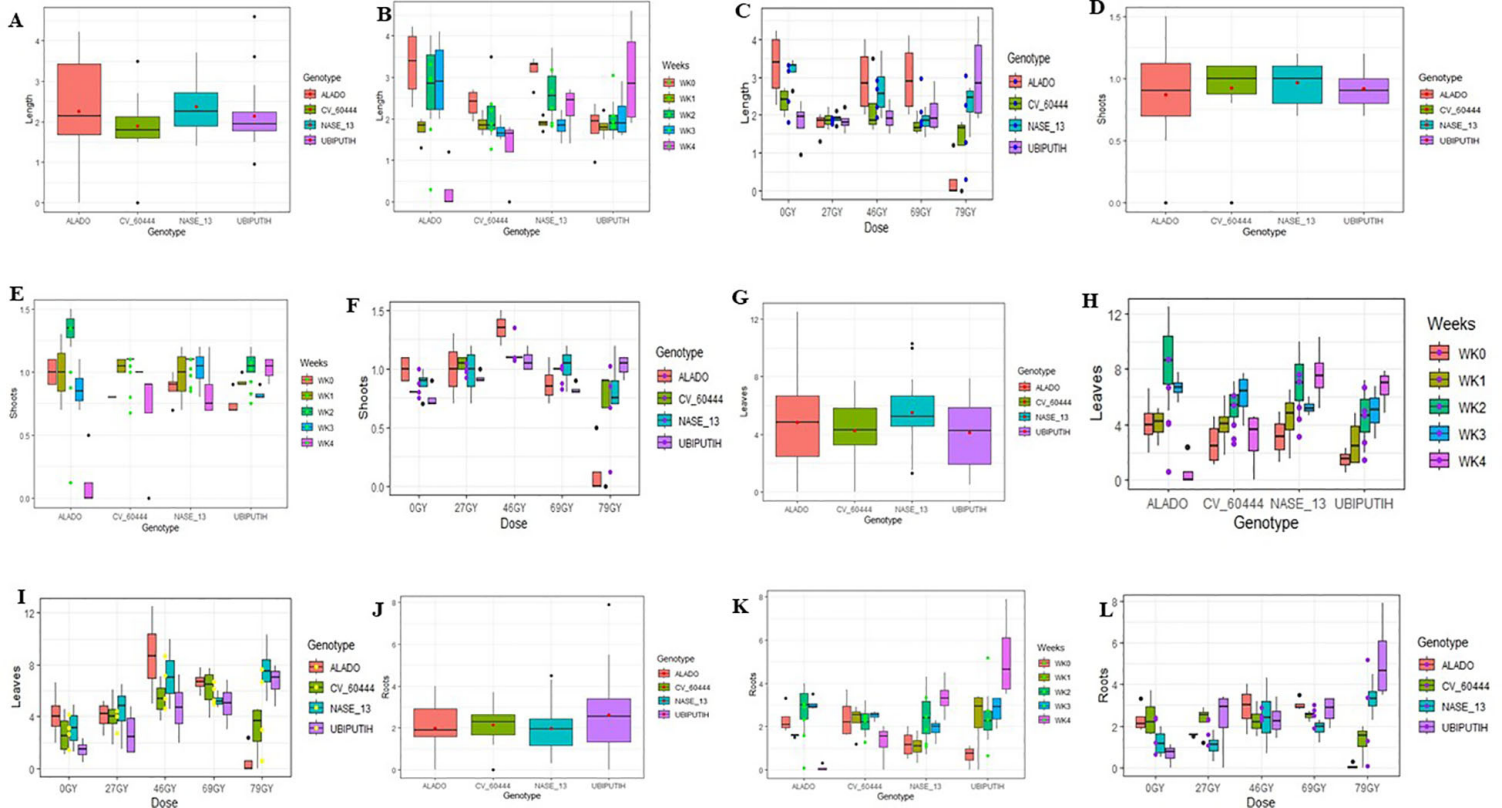
Mean Morphogenic Responses of ivNCs of Four Cassava Genotypes Following CGI Over a Four-Week Period. The data are presented as follows: **(A)** Average shoot length as influenced by genotype. **(B)** Interaction effects between genotype and time (weeks) on shoot length. **(C)** Interaction effects between genotype and CGI dose rates on shoot length. **(D)** Average number of shoots produced per genotype. **(E)** Interaction effects between genotype and time (weeks) on shoot count. **(F)** Interaction effects between genotype and CGI dose rates on shoot count. **(G)** Average number of leaves produced per genotype. **(H)** Interaction effects between genotype and time (weeks) on leaf production. **(I)** Interaction effects between genotype and CGI dose rates on leaf production. **(J)** Average number of roots produced per genotype. **(K)** Interaction effects between genotype and time (weeks) on root development. **(L)** Interaction effects between genotype and CGI dose rates on root development.

*Shoot Length Response:* Among the genotypes, NASE 13 recorded the highest mean shoot length (2.37 ± 0.32cm), followed by Alado (2.25 ± 0.47cm), CV_60444 (1.88 ± 0.28cm), and Ubi Putih (1.61 ± 0.3 cm). No significant differences were observed between genotypes (P = 0.23676, 95% CI) (**Figure 9A**). The genotypes responded differently over time, with Alado exhibiting the longest shoots at week three, NASE 13 at week two, CV_60444 at week two, and Ubi Putih at week four. Significant differences were observed in shoot length across weeks (P = 0.01353, 95% CI), and genotype-week interaction (P = 4.945 × 10^-5^, 99.999% CI) (**Figure 9B**). Genotype-dose interaction revealed optimal growth at varying doses: Alado at 69 Gy (3.00 ± 0.49cm), NASE 13 at 46 Gy (2.69 ± 0.39cm), CV_60444 at 46 Gy (2.20 ± 0.44cm), and Ubi Putih at 79Gy (3.02 ± 0.63cm) (**Figure 9C**). However, control plants (0 Gy) generally performed better than irradiated mutants, except for Ubi Putih (2.43 ± 0.49cm). No significant differences were observed for shoot length across doses (P = 0.1353, 95% CI), though genotype-dose interaction was significant (P = 4.945 × 10^-5^, 99.999% CI).

*Number of Shoots*: Mean shoot production varied across genotypes, with NASE 13 yielding the highest number (0.97 ± 0.14), followed by CV_60444 (0.93 ± 0.18), Ubi Putih (0.92 ± 0.16), and Alado (0.87 ± 0.28). No significant differences were observed among genotypes (P = 0.338, 95% CI) (**Figure 9D**). Genotype-week interaction showed Alado produced the highest number at week two, NASE 13 at week three, CV_60444 at week one, and Ubi Putih at week four. Significant differences were observed across weeks (P = 1.422 × 10^-5^, 99.999% CI) and genotype-week interaction (P = 6.057 × 10^-6^, 99.999% CI) (**Figure 9E**). Across doses, all genotypes performed best at 46 Gy, with Alado (1.35 ± 0.07), NASE 13 (1.10 ± 0.00), CV_60444 (1.10 ± 0.00), and Ubi Putih (1.08 ± 0.05) (**Figure 9F**). Significant differences were noted for dose response (P = 1.422 × 10^-5^, 99.999% CI) and genotype-dose interaction (P = 6.057 × 10^-6^, 99.999% CI).

*Number of Leaves:* Leaf production varied across genotypes, with NASE 13 producing the highest count (5.51 ± 0.58), followed by Alado (4.84 ± 0.74), CV_60444 (4.24 ± 0.52), and Ubi Putih (4.10 ± 0.60). Significant differences were observed across genotypes (P = 5.056 × 10^-4^, 99.999% CI) (**Figure 9G**). Genotypes showed varied responses over time, with Alado producing the highest number of leaves at week two, NASE 13 at week four, CV_60444 at week three, and Ubi Putih at week four. Significant differences were observed across weeks (P = 9.448 × 10^-3^, 99.99% CI) and genotype-week interaction (P = 4.993 × 10^-5^, 99.999% CI) (**Figure 9H**). Across doses, Alado at 46Gy (8.70 ± 1.59) exhibited the highest leaf count, followed by NASE 13 at 46Gy (7.63 ± 1.05), CV_60444 at 69Gy (6.13 ± 0.84), and Ubi Putih at 79 Gy (6.68 ± 0.64) (**Figure 9I**). Significant differences were noted across doses (P = 9.448 × 10^-3^, 99.99% CI) and genotype-dose interaction (P = 4.993 × 10^-5^, 99.999% CI).

*Number of Roots*: Root production varied across genotypes, with Ubi Putih exhibiting the highest count (2.65 ± 0.54), followed by CV_60444 (2.16 ± 0.29), NASE 13 (1.99 ± 0.41), and Alado (1.98 ± 0.46). Significant differences were observed across genotypes (P = 4.349 × 10^-2^, 95% CI) (**Figure 9J**). Weekly analysis revealed Ubi Putih and NASE 13 had peak root production at week four, CV_60444 at week two, and Alado at week two. No significant differences were observed across weeks (P = 0.121246, 95% CI), while genotype-week interaction was significant (P = 4.358 × 10^-6^, 99.999% CI) (**Figure 9K**). Across doses, Ubi Putih at 79 Gy (5.18 ± 1.00) performed best, followed by NASE 13 at 79 Gy (3.35 ± 0.45), CV_60444 at 69 Gy (3.03 ± 0.16), and CV_60444 at 79 Gy (2.50 ± 0.11). Control plants responded differently (CV_60444 at 0Gy (2.43 ± 0.49), Alado at 0 Gy (2.33 ± 0.34), NASE 13 at 0 Gy (1.20 ± 0.34), Ubi Putih at 0 Gy (0.65 ± 0.24)) (**Figure 9L**). No significant differences were observed across doses (P = 1.2125 × ^10-6^, 95% CI), though genotype-dose interaction was significant (P = 4.358 × 10^-6^, 99.999% CI).

CGIs significantly influenced shoot length, shoot count, leaf production, and root development across four cassava genotypes. 46Gy exhibited optimal growth across most parameters, while higher doses (69–79Gy) demonstrated mixed effects. Weekly progression revealed genotype-dependent responses, with some traits peaking at different time points. The findings underscore the need for dose optimization in cassava mutation breeding to enhance agronomic performance without compromising regeneration.

### Effects of CGI on SAMs in two cassava genotypes

The SAMs of the two cassava genotypes (Alado and NASE 13) responded differently to CGI producing shoots, leaves, roots, and varying shoot lengths. The accumulated effects of gamma radiation were tested at three doses 21, 27 and 31 Gy administered weekly for three weeks.

**Figure 10:**
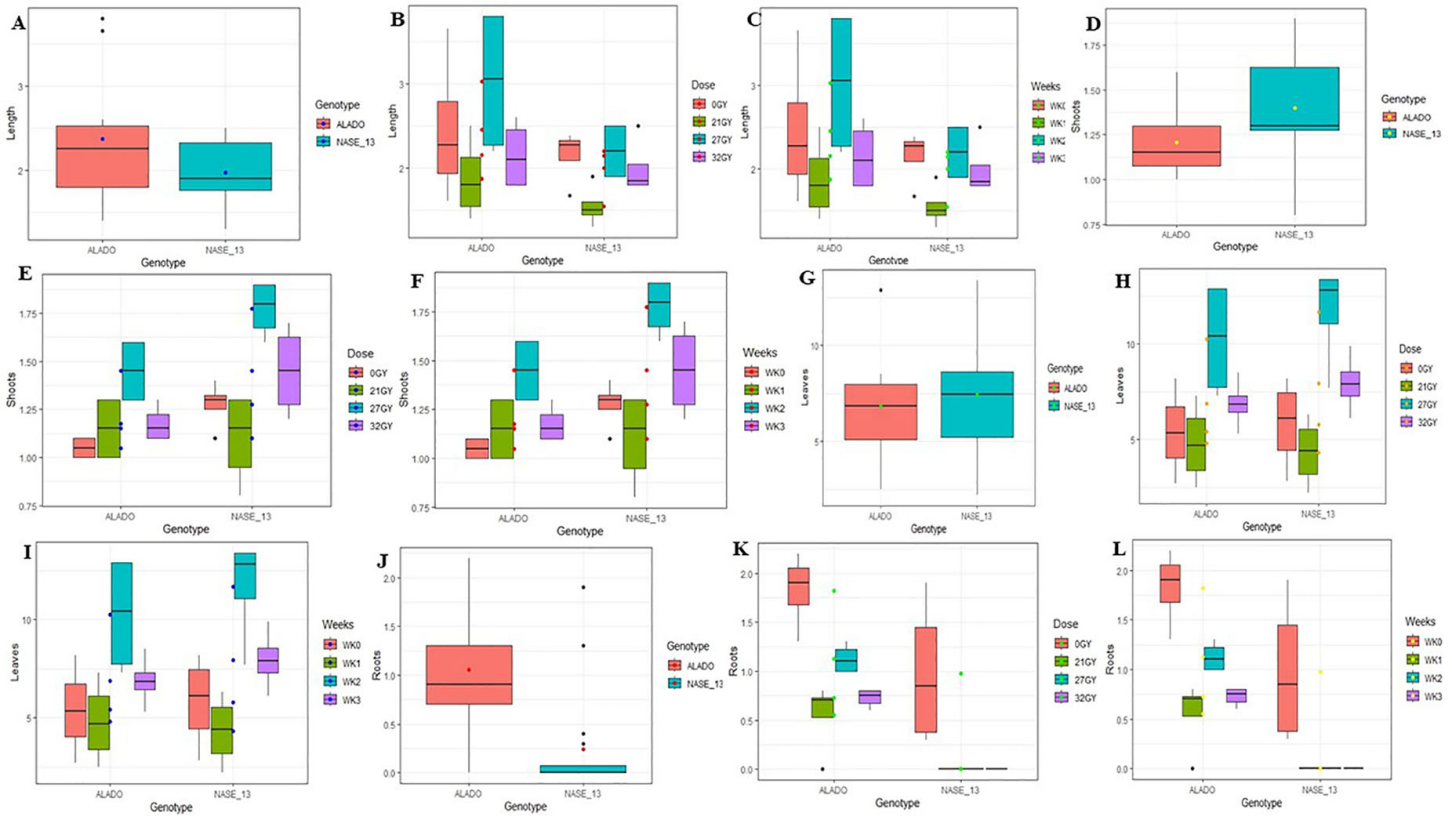
Mean Morphogenic Responses of SAMs of Two Cassava Genotypes Exposed to CGI Over a Three-Week Period. The data are presented as follows: **(A)** Average shoot length as influenced by genotype. **(B)** Interaction effects between genotype and CGI dose rates on shoot length. **(C)** Interaction effects between genotype and time (weeks) on shoot length. **(D)** Average number of shoots produced per genotype. **(E)** Interaction effects between genotype and CGI dose rates on shoot count. **(F)** Interaction effects between genotype and time (weeks) on shoot count. **(G)** Average number of leaves produced per genotype. **(H)** Interaction effects between genotype and CGI dose rates on leaf production. **(I)** Interaction effects between genotype and time (weeks) on leaf production. **(J)** Average number of roots produced per genotype. **(K)** Interaction effects between genotype and CGI dose rates on root development. **(L)** Interaction effects between genotype and time (weeks) on root development.

*Shoot Length:* After the FRS, Alado exhibited a greater average shoot length (2.378 ± 0.33cm) compared to NASE 13 (1.975 ± 0.25cm), with significant differences observed between genotypes (P = 4.655 × 10^-2^, 95% CI; **Figure 10A**). Both genotypes showed improved growth at 27 Gy, where Alado (3.03 ± 0.45cm) outperformed NASE 13 (2.20 ± 0.17cm). Significant differences were observed for dose (P = 7.331 × 10^-3^, 95% CI) and genotype-dose interaction (P = 3.95 × 10^-1^, 95% CI; **Figure 9B**). Although shoot length differed across weeks, no significant differences were detected (P = 7.331 × 10^-3^, 95% CI; **Figure 10C**).

*Number of Shoots:* Following FRS, NASE 13 (1.4 ± 0.25) produced more shoots than Alado (1.21 ± 0.19), with significant differences observed (P = 2.687 × 10^-2^, 95% CI; **Figure 10D**). Both genotypes performed best at 27 Gy, with NASE 13 (1.77 ± 0.08) exceeding results from 31 Gy (1.45 ± 0.12) and 21 Gy (1.10 ± 0.12). Alado also showed optimal growth at 27 Gy (1.45 ± 0.09). Significant differences were found for dose (P = 2.209 × 10^-4^, 99.999% CI), but genotype-dose interaction was not significant (P = 1.202 × 10^-1^, 95% CI; **Figure 10E**). No significant differences were noted across weeks (P = 2.209 × 10^-4^, 99.99% CI; **Figure 10F**).

*Number of Leaves:* NASE 13 (7.44 ± 0.83) produced more leaves than Alado (6.83 ± 0.09), although differences were not significant (P = 4.459 × 10^-1^, 95% CI; **Figure 10G**). Optimal leaf production occurred at 27 Gy, with NASE 13 (11.68 ± 1.35) outperforming its performance at 31 Gy (7.95 ± 0.78) and 21 Gy (4.33 ± 0.91). Alado followed a similar trend. Significant differences were found for dose (P = 3.156 × 10^-5^, 99.999% CI), but genotype-dose interaction was not significant (P = 6.593 × 10^-1^, 95% CI; **Figure 10H**). No significant differences were observed across weeks (P = 6.784 × 10^-5^, 99.999% CI; **Figure 10I**).

*Number of Roots:* Alado (1.06 ± 0.35) produced significantly more roots than NASE 13 (0.25 ± 0.33), with strong significance (P = 7.554 × 10^-9^, 99.999% CI; **Figure 10J**). Alado’s root production was highest at 27 Gy (1.13 ± 0.08), though it was lower than the control at 0 Gy (1.83 ± 0.19). NASE 13 did not produce roots at any radiation dose. Significant differences were found for dose (P = 3.274 × 10^-2^, 95% CI) and genotype-dose interaction (P = 8.982 × 10^-3^, 99.99% CI; **Figure 10K**). Alado responded best in week two, whereas NASE 13 showed no root development. Significant differences were noted across weeks (P = 3.274 × 10^-2^, 99.99% CI; **Figure 10L**).

### Determination of growth reduction dose in cassava tissue types following AGI

This study assessed the growth reduction doses (GR_50_) for different tissue types: *iv*NCs, SAMs, and FEC of Ugandan cassava genotypes exposed to AGI.

### Determination of GR_50_ ivNCs of Ugandan cassava genotypes exposed to AGI

**Figure 11:**
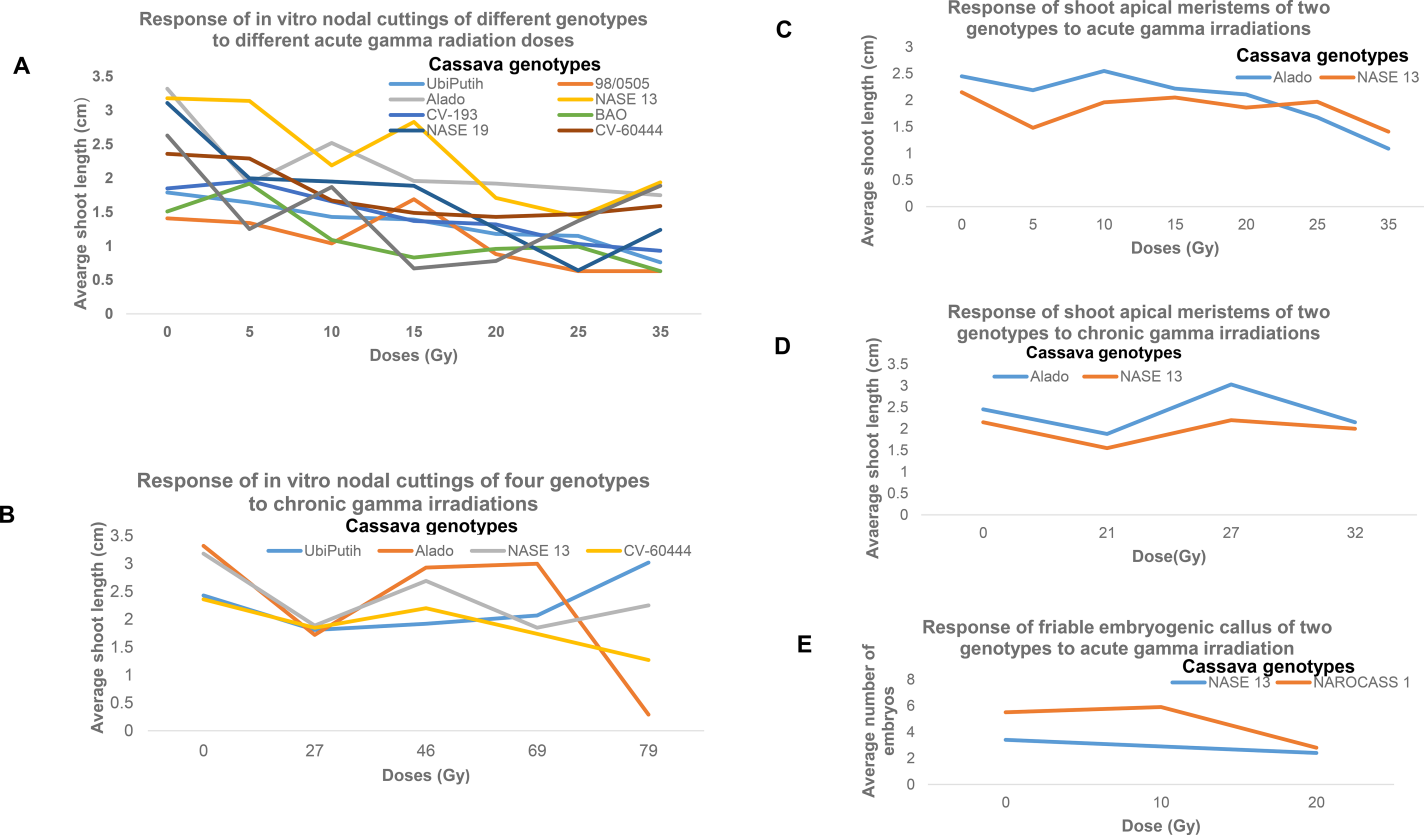
The response of different tissue types from different cassava genotypes due to their exposure to either acute or chronic gamma irradiations. **(A)** Response of ivNCs to acute gamma irradiations, **(B)** Response of ivNCs to chronic gamma irradiations, **(C)** Response of SAMs to acute gamma irradiations, **(D)** Response of SAMs to chronic gamma irradiations and **(E)** Response of FEC to acute gamma irradiations.

The average shoot length of the regenerated ivNCs of the eight Ugandan cassava genotypes and the control Ubi Putih, in relation to the different radiation doses (0, 5, 10, 15, 20, 25, and 35 Gy) was used to calculate the GR_50_ values. Based on the data collected, the GR_50_ was calculated for CV_193, NAROCASS 1, Ubi Putih, NASE 13, BAO, 98/0505, CV-60444, Alado and NASE 19 were 32, 27, 31, 32, 29, 31, 39, 31 and 19 Gy, respectively. The R-squared value of 0.7 or higher explains a significant proportion of variability in the dependent variable explained by the independent variable. The R-squared values for the genotypes were within the acceptable range of 0.5 to 1 with the exception of NAROCASS1 (R^2^ = 0.2682). The highest R-Squared values was obtained in Ubi Putih (0.9901), followed by CV_193 (0.9595), then NASE 19 (0.8371), BAO (0.8077), NASE 13 (0.7858), CV_60444 (0.7597), 98/0505 (0.7436) and Alado (0.7246) (**Table 2**; ; **Figure 11A**).

**Table 2:** Regression parameters and GR_50_ Estimates for cassava genotypes exposed to acute and chronic gamma irradiation.

Radiation type	Genotype	Mean values (cm)	R^2^ value	Slope	Calculated dose
**Acute ivNCs**	Alado	2.18 ± 0.52	0.5251	-0.0338	30.99
	98/0505	1.09 ± 0.38	0.553	-0.0251	31.00
	NASE 13	2.35 ± 0.65	0.6174	-0.046	32.16
	BAO	1.13 ± 0.41	0.6524	-0.0295	28.49
	CV-193	1.45 ± 0.37	0.9208	-0.0314	32.31
	NASE 19	1.73 ± 0.73	0.7008	-0.0545	18.88
	CV-60444	1.76 ± 0.37	0.5771	-0.025	38.80
	NAROCASS 1	1.49 ± 0.64	0.0719	-0.0153	27.48
	Ubi Puth	1.33 ± 0.32	0.9804	-0.0281	31.34
**Acute-SAMs**	Alado	2.04 ± 0.47	0.7953	-0.0372	27.89
	NASE 13	1.84 ± 0.26	0.1848	-0.0102	90.25
**Acute-FEC**	NASE 13	2.90 ± 0.41	1.0000	-0.05	34.00
	NAROCASS 1	4.73 ± 1.38	0.641	-0.135	24.69
**Chronic-ivNCs**	Alado	2.25 ± 1.12	0.292	-0.0212	72.20
	NASE 13	2.37 ± 0.50	0.3734	-0.0108	116.6
	CV-60444	1.88 ± 0.38	0.6791	-0.011	108.2
	Ubi Putih	2.25 ± 0.44	0.1164	0.0052	-154.54
**Chronic-SAMs**	Alado	2.38 ± 0.43	0.027	-0.0002	5781
	NASE 13	1.98 ± 0.26	3 x 10^-5^	-0.0035	276.9

### Determination of GR_50_ of SAMs for the two Ugandan cassava genotypes to AGI

**Figure 12:**
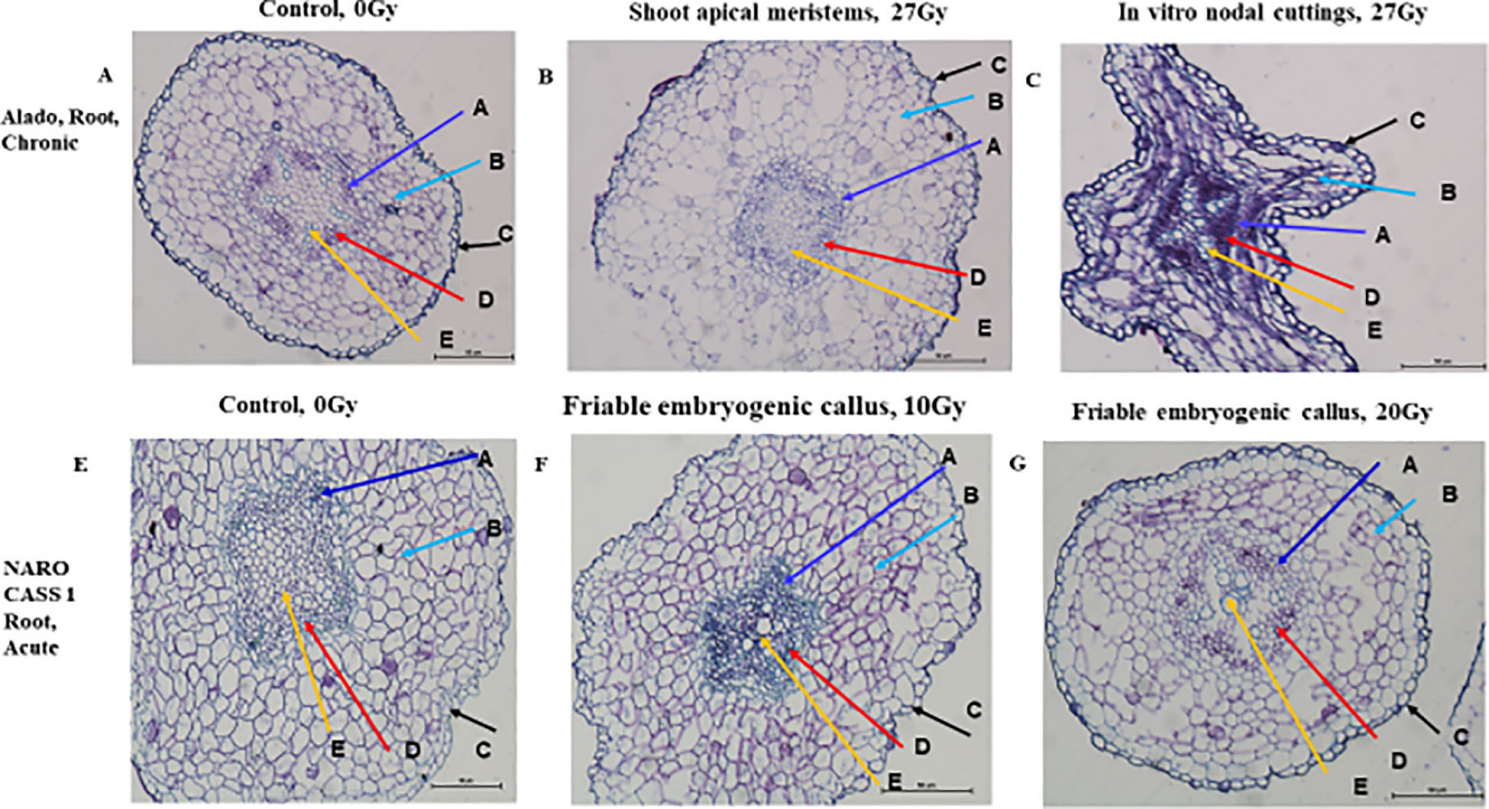
Histological Illustration of Dicotyledonous Root Tissues in Cassava Genotypes Exposed to Gamma Irradiation. Microscopic images (×20 magnification) depict structural changes in dicotyledonous root tissues of cassava genotypes Alado and NAROCASS 1 following exposure to either acute gamma irradiation (AGI) or chronic gamma irradiation (CGI). Comparative observations were made across two tissue type in vitro nodular cultures (ivNCs) and shoot apical meristems (SAMs) to assess irradiation-induced anatomical modifications. Each pictorial panel is labeled to highlight key tissue regions: **(A)** Alado, Root, Chronic, Control, 0Gy, **(B)** Alado, Root, Chronic, Shoot aprical meristems, 27Gy, **(C)** Alado, Root, Chronic, *In vitro* nodal cuttings, 27Gy, **(E)** NAROCASS 1 root, acute, Control, 0Gy, **(F)** NAROCASS 1 root, acute, Friable embryogenic callus, 10Gy, **(G)** NAROCASS 1 root, acute, Friable embyrogenic callus, 20Gy.

The average shoot length of the regenerated SAMs of the two Ugandan cassava genotypes in relation to the different radiation doses (0, 5, 10, 15, 20, 25, and 35 Gy) was used to calculate the GR_50_ values. Alado (2.04 ± 0.47cm) had the highest average shoot length while NASE 13 had (1.84 ± 0.26cm) (**Table 2**). Based on the data collected, the GR_50_ was calculated for NASE 13 and Alado were 91 and 38 Gy, respectively (**Figure 12A, B**; **Table 2**). The R-squared values for the genotype Alado was within the acceptable of 0.5 to 1 (0.8918) unlike NASE 13 (R^2^ = 0.4299) whose value was so low indicating low variability in relation to the dependent and independent variables (**Table 2**). A negative slope was obtained given that as the dose increases, the shoot length of the plant decreased, indicating that the rate of change of one variable with respect to another had inverse relationship (**Table 2**; **Figure 11C**).

### Determination of GR_50_ of FEC for the two Ugandan cassava genotypes of AGI

The mean number of embryo from the regenerated FEC of the two Ugandan cassava genotypes in relation to the different radiation doses (0, 10, and 20) Gy was used calculate the GR_50_ values. NAROCASS 1 (4.73 ± 1.38) had the highest average number of embryos while NASE 13 (2.90 ± 0.41) had the least. Based on the data collected, the GR_50_ calculated for NASE 13 and NAROCASS1 were 34 and 24.69 Gy respectively (**Table 2**). The R-squared values for the genotypes NASE 13 and NAROCASS1 was 1 and (0.641) respectively (**Table 2**). A negative downward slope was obtained implying that as the dose was increasing the shoot length of the plant decreased, indicating that the rate of change of one variable with respect to another has an inverse relationship (**Table 2**; **Figure 11E**). This analysis provides insights into genotype-specific radiosensitivity of cassava tissue types. While NASE 13 showed minimal response in SAM regeneration, *iv*NC and FEC exhibited more pronounced dose-dependent effects, supporting dose optimization in cassava mutation breeding for improved tissue regeneration.

### Determination of GR_50_ in cassava tissue types (ivNCs and SAMs) exposed to chronic gamma irradiation

This study assessed the growth reduction doses (GR_50_) for *iv*NCs and SAMs of Ugandan cassava genotypes subjected to CGI at varying doses.

### Determination of GR_50_ of *iv*NCs for the three Ugandan cassava genotypes and control exposed to CGIs

The average shoot length of the regenerated *iv*NCs of the three Ugandan cassava genotypes and control in relation to the different radiation doses (0, 27, 46, 69 and 79 Gy) was used to calculate the GR_50_ values. NASE 13 (2.37 ± 0.50 cm) had the highest average shoot length followed by Alado (2.25 ± 1.12 cm), then Ubi Puith (2.25 ± 0.44 cm) and least CV-60444 (1.88 ± 0.38cm) (**Table 2**). Based on the data collected, the GR_50_ was calculated for Alado, NASE 13, CV-60444 and Ubi Putih were 72, 117, 108 and154 respectively (**Table 2**). The R-squared values for the genotype CV_60444 (R^2^ = 0.8241), NASE 13 (R^2^ = 0.611), Alado (R^2^ = 0.5403) and Ubi Putih (R^2^ = 0.3412) (**Table 2**). This analysis highlights genotype-specific responses to CGI with CV_60444 showing the highest radio sensitivity (GR_50_ = 154 Gy), while Alado exhibited the least radio sensitivity. #A negative slope was obtained for Alado, NASE 13, CV-60444 with the exception of Ubi Putih which had a positive slope. The negative slope downwards implies that as the dose was increasing, the shoot length of the plant decreased (**Table 2**; **Figure 11B**) while for Ubi Putih as the dose increased, the shootlength increased (**Table 2**; **Figure 11B**). The findings underscore the importance of optimized irradiation protocols for cassava breeding programs targeting enhanced tissue regeneration.

### Determination of GR_50_ of SAMs for the two Ugandan cassava genotypes to CGIs

The average shoot length of the regenerated SAMs of the two Ugandan cassava genotypes exposed to CGIs (0, 21, 27 and 31 Gy) was used to calculate the GR_50_ values. Based on the data collected, the GR_50_ resulted in a large calculated dose for Alado (5781 Gy). The slope was negative downwards implying that as the dose was increasing the shoot length of the plant decreased, indicating that the rate of change of one variable with respect to another is small while for NASE 13 the calculated dose was (276.9 Gy) (**Table 2**; **Figure 11D**). The R-squared values for the genotype Alado (R^2^ = 0.005) and NASE 13 (R^2^ = 0.1642) (**Table 2**). The absence of a significant dose-response relationship suggests that SAM regeneration in Alado and NASE 13 was not markedly affected by chronic gamma irradiation at the tested doses. Further studies with higher doses or alternative irradiation protocols may be required to assess their radiosensitivity more effectively.

### Histological observation of the cross-section of the mutant lines and controls under a transmission electronic microscopy

A selection of mutant lines and control samples from five cassava genotypes (Alado, NAROCASS 1, NASE 19, CV_193, and CV_60444) and ivNCs, SAMs, and FEC were analyzed histologically using hematoxylin and eosin staining.

#### Staining and tissue structure analysis

Hematoxylin selectively binds to nucleic acids in the cell nucleus, staining it blue or purple, while eosin stains proteins non-specifically, marking other cell structures and connective tissues in shades of orange-pink to red. The cross-section of roots and shoots in different tissue types (ivNCs, SAMs, and FEC) consistently exhibited blue/purple staining, indicating active cellular processes. Structural similarities were observed across root cells of control plants, SAMs, ivNCs and FEC suggesting similar organelle formation during developmental stages (**Figures 12A–E**). The impact of CGI was evident in the epidermis and cellular contents of Alado’s root cells (**Figure 12C**), whereas control tissue and SAMs exposed to 27Gy showed no deformations, unlike ivNCs, which exhibited noticeable alterations (**Figures 12A, B**).

#### Effects of AGI

**Figure 13:**
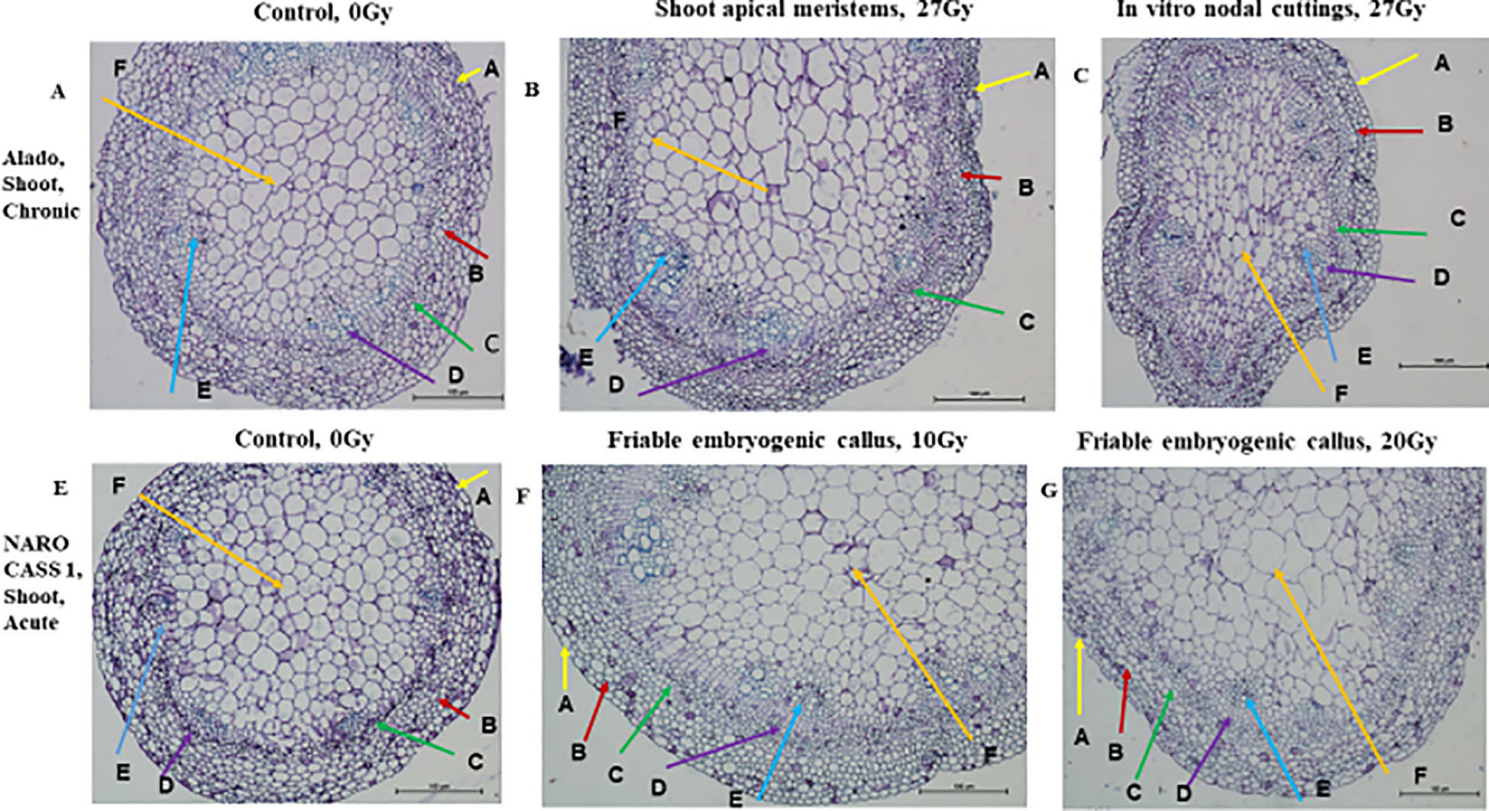
Histological Illustration of Dicotyledonous Shoot Tissues in Cassava Genotypes Exposed to Gamma Irradiation. Microscopic images (×10 magnification) depict structural changes in shoot tissues of cassava genotypes Alado and NAROCASS 1 following exposure to either acute gamma irradiation (AGI) or chronic gamma irradiation (CGI). Tissue samples were derived from both in vitro nodular cultures (ivNCs) and shoot apical meristems (SAMs) to assess irradiation-induced anatomical modifications. Each pictorial panel is labeled to highlight key anatomical regions: **(A)** Alado, Root, Chronic, Control, 0Gy, **(B)** Alado, Root, Chronic, Shoot aprical meristems, 27Gy, **(C)** Alado, Root, Chronic, *In vitro* nodal cuttings, 27Gy, **(E)** NAROCASS 1 root, acute, Control, 0Gy, **(F)** NAROCASS 1 root, acute, Friable embryogenic callus, 10Gy, **(G)** NAROCASS 1 root, acute, Friable embyrogenic callus, 20Gy.

Roots exposed to acute gamma doses (10 Gy and 20 Gy) showed no morphological differences compared to controls (**Figures 12E–G**). No significant changes were observed in shoot cell structures across irradiated and control tissue types (ivNCs, SAMs, and FEC), despite varying radiation doses (**Figures 13A–F**). All shoot cells exposed to (ACGI) exhibited cell enlargement within the pith, a key region for water and nutrient storage (**Figures 13A–F**).

Histological observations indicate that while gamma irradiation influences epidermal integrity and internal cell morphology, core structural elements remain largely unaffected across tissue types. Cell enlargement in shoot pith regions suggests a potential physiological adaptation to irradiation stress, warranting further investigation into functional implications for cassava growth and resilience.

### Discussion of results

Cassava (*Manihot esculenta* Crantz) is a highly resilient crop, capable of withstanding harsh climatic conditions, yet it faces numerous biotic and abiotic challenges, particularly viral diseases such as cassava brown streak disease (CBSD). To expand the genetic diversity of cassava and improve its resistance to viral pathogens, this study investigated the effects of ionizing gamma irradiation, focusing on its impact on the regenerative ability of FEC, SAMs, and *iv*NCs.

Gamma irradiation, emitted by Cobalt-60 (Co-60) or Cesium-137 (Cs-137) radionuclides, can induce diverse responses at cellular, tissue, organ, and whole plant levels, leading to either survival or cell death (Jan et al., 2012). Earlier studies utilizing a Co-60 commercial radiation machine with doses of 5 to 25 Gy demonstrated that 5–15 Gy was optimal for ivNC regeneration, whereas FEC tissues failed to survive (Apio et al., 2024). Building upon this, the present study utilized the GM 8000 Biobeam radiation machine for AGI and the chronic gamma greenhouse (CGGH) for CGI exposure, both powered by a Cs-137 pencil source.

#### Mechanisms of gamma irradiation and tissue responses

Gamma irradiation exerts its biological effects on plant tissues through two principal pathways:

Direct interactions, which involve ionizing damage to cellular macromolecules, particularly DNA, resulting in strand breaks, chromosomal aberrations, and genomic instability. Indirect interactions, mediated by the radiolysis of water molecules, leading to the generation of reactive oxygen species (ROS) such as hydroxyl radicals (•OH), hydrogen peroxide (H_2_O_2_), and superoxide anions (O^2-^). These ROS induce oxidative stress, disrupting cellular homeostasis and altering plant morphology, physiology, and biochemical pathways (Choi et al., 2021). The cumulative impact of these radiation-induced perturbations fosters genotypic variability, which can be harnessed for the selection of improved cassava lines exhibiting enhanced agronomic traits, stress tolerance, or disease resistance. This mechanism underpins mutation breeding strategies aimed at accelerating crop improvement and genetic diversification.

#### Cassava tissue responses to acute gamma irradiation

This study presents the first documented evaluation of regenerative responses in *in vitro* nodal cuttings (ivNCs), shoot apical meristems (SAMs), and friable embryogenic calli (FEC) derived from selected Ugandan cassava genotypes under acute gamma irradiation (AGI). The optimal radiation doses for regeneration were determined as 5-15Gy for ivNCs across nine genotypes (**Figures 4A–D**) and SAMs across two genotypes for shoot length (**Figure 6B**), shoots (**Figure 6E**), leaves (**Figure 6H**) and roots (**Figure 6K**). Doses ranging from 20-35 Gy were found to inhibit regeneration in ivNCs (**Figure 6K**) and SAMs (**Figures 4C, D**) entirely, resulting in complete loss of plant viability at the first regeneration stage (FRS). Notably, FEC tissues from NAROCASS 1 successfully regenerated into whole plants at 10 and 20 Gy (**Figures 8A–Q**).

The ability of these tissues to regenerate at lower doses suggests efficient integration of single base substitutions (SBS) into the plant genome, allowing continued growth and development. This mechanism aligns with previous observations in Arabidopsis thaliana, where SBS events facilitated radiation-induced mutagenesis and regeneration (Hase et al., 2020). Moreover, the capacity of certain genotypes to withstand oxidative stress at 5–15 Gy indicates activation of intrinsic antioxidant defense systems. Enzymes such as superoxide dismutase (SOD), catalase (CAT), ascorbate peroxidase (APX), and peroxidase (POD) likely play pivotal roles in mitigating the damaging effects of reactive oxygen species (ROS), thereby preserving cellular integrity and promoting normal plant growth (Hong et al., 2018).

### Cassava tissue responses to chronic gamma irradiation

Chronic gamma irradiation (CGI) facilitates gradual mutation accumulation, often at lower doses than acute exposure, due to its extended delivery over time. This study represents the first reported analysis of CGI effects on shoot apical meristems (SAMs) and *in vitro* nodal cuttings (ivNCs) in Ugandan cassava genotypes. The trends indicate a gradual increase in the different parameters measured with time from week 1 to week 3 and toppling off at week 4 for inNCs (**Figures 5A–D**) while SAMs a similar noticeable trend was observed (**Figures 6C, F, I, L**). The ivNCs exhibited optimal regeneration at 46 Gy, followed by 69 Gy, 27 Gy, and least at 79 Gy (**Figure 9**). This was evident in the parameters measured: Shoot length (**Figure 9C**), Shoots (**Figure 9F**), Leaves (**Figure 9I**) and Roots (**Figure 9L**). In contrast, SAM regeneration peaked at 27 Gy, followed by 31 Gy, with the lowest regeneration observed at 21 Gy (**Figure 10B**). This was demonstrated in shoot length (**Figure 10B**), Shoots (**Figure 10E**), Leaves (**Figure 10E**) and Roots (**Figure 10K**). These dose-dependent responses highlight tissue-specific radiosensitivity and suggest differential thresholds for mutation tolerance and repair. Plants exposed to CGI over three- and four-week durations demonstrated significant leaf, shoot, and root formation, with notably increased shoot length at the FRS. Although CGI typically produces fewer reactive oxygen species (ROS) than acute irradiation, it can induce homozygous deletions exceeding 50 base pairs, contributing to stable genetic modifications (Hase et al., 2020). The complete loss of tissue viability at 79 Gy is likely attributable to cumulative and irreparable DNA damage, resulting in genomic instability and cell death. This phenomenon aligns with prior findings on radiation-induced lethality and chromosomal fragmentation (Chatterjee and Walker, 2017; Szurman-Zubrzycka et al., 2023).

### Impact of gamma irradiation on chromosomal integrity

Both acute and chronic gamma irradiation (ACGI) induced a spectrum of genomic alterations in cassava tissues, including deletions, duplications, translocations, and inversions. These structural changes influenced the regenerative responses of different tissue types and genotypes, underscoring the genotype-dependent nature of radiation tolerance (Chatterjee and Walker, 2017). Acute gamma doses ranging from 5–15 Gy promoted successful regeneration of shoot apical meristems (SAMs) (**Figures 6B, E, H, K**) and *in vitro* nodal cuttings (ivNCs) (**Figures 4A–D**), while 10 Gy and 20 Gy supported friable embryogenic callus (FEC) regeneration in NAROCASS 1 (**Figure 7B**). In contrast, chronic gamma doses of 27, 46, and 69 Gy for ivNCs (**Figures 9C, F, I, L**), and 27 and 32 Gy for SAMs (**Figures 10B, E, H, K**), enabled tissue growth at the FRS, suggesting a differential threshold for mutation accumulation and repair under prolonged exposure.

The ability of cassava tissues to recover from radiation-induced genomic lesions implies activation of cell cycle checkpoints and engagement of DNA damage response (DDR) pathways. These mechanisms likely initiate targeted repair processes, including the deployment of translesion synthesis (TLS) polymerases, which facilitate replication across damaged DNA templates. Such bypass systems ensure continuity of cell division and contribute to the maintenance of genetic stability despite mutagenic stress (Chatterjee and Walker, 2017; Szurman-Zubrzycka et al., 2023).

### Genotypic variability in gamma irradiation response

Genotypic differences played a pivotal role in determining tissue responses to gamma irradiation. When ivNCs were used, genotypes like BAO and 98/0505, had a low survival rate after the irradiation process, while Alado, CV-60444, NAROCASS 1 and NASE 19 demonstrated notable resilience (**Figures 3A–D**). With the use of SAMs, Alado showed remarkable shoot length and roots (**Figures 6A, J**) in comparison to NASE 13 which demonstrated the same for shoots and leaves (**Figures 6D, G**). A similar trend was observed with FEC, NAROCASS 1 performed best while NASE 13 was low (**Figure 7A**). Time in weeks was crucial for the response of the different genotypes. The increase in the parameters measured increased from week one to three and a decline at week 4 (**Figures 10C, F, I, L**). These observations reinforce the hypothesis that genetic constitution significantly influences radiosensitivity, dictating the capacity of plant tissues to repair radiation-induced mutations and maintain physiological integrity. The successful regeneration of shoot apical meristems (SAMs), in vitro nodal cuttings (ivNCs), and friable embryogenic calli (FEC) at the FRS suggests the presence of key proteins required for cell division. These include growth factors, growth factor receptors, signal transducers, and transcription factors, which collectively facilitate cellular proliferation and tissue morphogenesis (Inzé and De Veylder, 2006; Qi and Zhang, 2020).

In plants, the phytohormones auxins and cytokinins are central to regulating growth and developmental processes. In this study, NAA and BAP at 1 mg/ml were used to stimulate regeneration in SAMs and ivNCs of the Malaysian cassava variety Ubi Putih, as reported by the Malaysian Nuclear Agency (Nooralzina Noordin, personal communication). Previous research has shown that NAA and BAP enhance SAM regeneration in selected Ugandan cassava genotypes, whereas ivNCs did not require exogenous auxins or cytokinins for growth (Apio et al., 2021). Due to the antagonistic interaction between auxins and cytokinins, their concentration balance is critical in modulating plant development. This hormonal interplay influences lateral root formation, photosynthetic product accumulation, and chloroplast regulation, all of which are essential for post-irradiation recovery and sustained growth (Sosnowski et al., 2023).

### Regeneration of friable embryogenic calli

Friable embryogenic calli (FEC) consist of proliferating totipotent single cells capable of regenerating into complete plants. The developmental trajectory of FEC typically progresses through distinct morphological stages, globular, heart-shaped, torpedo-shaped, cotyledonary, and culminates in the maturation of plantlets. These transitions are tightly regulated by the application of auxins and cytokinins, which serve as key hormonal triggers for embryogenesis and morphogenesis. In this study, supplementation with 3 mg/ml NAA and 0.6 mg/ml BAP effectively promoted germination and maturation of mutant FEC. Subsequent reductions in hormone concentrations facilitated the formation of fully developed plants with robust root systems, indicating a successful transition from embryogenic callus to complete plantlets (**Figures 8N-Q**). Mutant FEC regeneration was successfully achieved in the genotype NAROCASS 1, whereas non-mutant FEC regeneration in selected Ugandan cassava genotypes was optimized using 1 µM/ml NAA, as previously reported (Apio et al., 2015). The regeneration process spanned approximately six months and required continuous sub-culturing to support progressive tissue differentiation and plantlet development.

Emerging evidence suggests that cell-cell communication in plant tissues is mediated by cell surface-bound receptors and signal transducers, which coordinate the spatial and temporal activation of developmental programs. These signaling networks enable competent embryos to undergo orderly transformation into whole plants, with auxins and cytokinins acting as central regulators of gene expression, cell division, and tissue patterning (Armingol et al., 2021).

### Physiological processes driving growth and development

Plant growth and development are fundamentally dependent on photosynthesis and transpiration, which facilitate tissue vascularization, nutrient transport, and the formation of key morphological structures such as leaves, shoots, and roots (Apio et al., 2021; Sosnowski et al., 2023). These physiological processes are tightly coupled with cellular energy metabolism, particularly the availability and turnover of adenosine triphosphate (ATP) and adenosine diphosphate (ADP). These molecules regulate intracellular energy flow, ensuring metabolic balance and supporting biosynthetic activities essential for growth. ATP and ADP are central to the Calvin cycle, which governs carbon dioxide fixation via the enzyme ribulose-1, 5-bisphosphate carboxylase/oxygenase (RuBisCO). This enzymatic pathway directly influences photosynthetic efficiency and, by extension, biomass accumulation and developmental progression (Cardol et al., 2003; Vera-Vives et al., 2024).

However, experimental data from this study reveal that not all irradiated mutants were capable of regeneration. Specifically, SAMs and ivNCs exposed to 20–35 Gy, along with FEC derived from NASE 13, failed to survive. This outcome suggests that ATP synthesis via cyclic electron flow may have been disrupted, impairing mitochondrial function and leading to energy deficits that culminated in tissue degeneration and growth arrest. Comparable disruptions in ATP synthesis have been documented in mutants of *Chlamydomonas reinhardtii*, where compromised energy metabolism adversely affects cellular viability and developmental outcomes (Cardol et al., 2003; Choi et al., 2021). These findings underscore the critical role of energy homeostasis in supporting regeneration and highlight the vulnerability of certain genotypes to radiation-induced metabolic stress.

### Impact of gamma irradiation on plant cell histomorphology

Histological analysis was performed on selected mutant lines representing various tissue types subjected to acute and chronic gamma irradiation (ACGI). The staining protocol employed hematoxylin and eosin (H&E), where hematoxylin binds specifically to nucleic acids, imparting a purple-blue coloration, while eosin non-specifically stains proteins, producing a pink hue. The observed purple-blue coloration in stained tissues confirmed the presence of deoxyribonucleic acid (DNA) and ribonucleic acid (RNA), indicating that the cells remained viable and their organelles were largely intact post-irradiation. This suggests that the irradiation did not compromise the fundamental cellular architecture required for growth and development (Minchin and Lodge, 2019). Conversely, the absence of pink coloration implies minimal protein synthesis activity, likely due to reduced transcription of DNA into RNA and limited translation of RNA into proteins. This outcome suggests that while nucleic acids were preserved, the metabolic machinery responsible for protein biosynthesis may have been downregulated or temporarily suppressed (Minchin and Lodge, 2019).

Histomorphological examination of both shoot and root tissues revealed that key internal structures—including xylem, phloem, parenchyma, and sclerenchyma cells, remained unaffected by irradiation. Their structural integrity supports continued functionality, nutrient transport, and mechanical support, all of which are essential for sustained plant growth (**Figures 12**, **13**). However, some anomalies were noted. In **Figure 12B**, epidermal rupture was observed, likely due to mechanical stress during tissue processing. In **Figure 12C**, epidermal deformation was evident and may be attributed to the effects of high-dose chronic gamma irradiation (CGI) on the epidermis and cortex of the Alado genotype (Choi et al., 2021). These localized disruptions underscore the dose-dependent sensitivity of surface tissues to prolonged radiation exposure. .

## Conclusion

This study provides novel insights into the radiosensitivity of Ugandan cassava genotypes, establishing foundational data for mutation breeding applications. The findings underscore the importance of dose optimization, which ensures a balance between effective mutation induction and tissue viability. This balance is critical for facilitating successful regeneration of cassava tissues in breeding programs aimed at enhancing resilience to both abiotic and biotic stresses.

Gamma irradiation was shown to influence cassava tissue regeneration in a genotype- and dose-dependent manner. Acute gamma irradiation (AGI) supported optimal regeneration at 5–15 Gy for shoot apical meristems (SAMs) and *in vitro* nodal cuttings (ivNCs), and at 10–20 Gy for friable embryogenic calli (FEC), promoting tissue survival and growth. Chronic gamma irradiation (CGI) facilitated regeneration at 46 Gy for ivNCs and 27 Gy for SAMs, sustaining tissue development. FEC regeneration was successful in NAROCASS 1 at both 10 Gy and 20 Gy, with greater embryo numbers observed at 20 Gy. ivNC regeneration was feasible for seven genotypes, excluding BAO and 98/0505, which exhibited poor responses. Under CGI, Alado, CV-60444, and Ubi Putih regenerated successfully, regardless of tissue type or exposure duration, while NASE 13 demonstrated limited regenerative capacity.

These findings highlight the potential of gamma-irradiated *in vitro*-derived cassava tissues to regenerate into whole mutant plants, offering a robust platform for cassava mutation breeding. Future research should prioritize evaluating the adaptation and resistance of regenerated mutant lines to key viral diseases, particularly cassava brown streak disease (CBSD) and cassava mosaic disease (CMD). Additionally, the study emphasizes the critical roles of auxin-cytokinin balance, ATP-dependent energy metabolism, and cell signaling mechanisms in regulating cassava tissue regeneration under gamma irradiation stress. These insights contribute to the refinement of tissue culture protocols, the selection of resilient genotypes, and the advancement of cassava improvement strategies through mutation breeding.

## Data Availability

The original contributions presented in the study are included in the article/supplementary material. Further inquiries can be directed to the corresponding author.
